# General Framework for the Optimization of the Human-Robot Collaboration Decision-Making Process Through the Ability to Change Performance Metrics

**DOI:** 10.3389/frobt.2021.736644

**Published:** 2021-10-25

**Authors:** Mélodie Hani Daniel Zakaria, Sébastien Lengagne, Juan Antonio Corrales Ramón, Youcef Mezouar

**Affiliations:** ^1^ CNRS, Clermont Auvergne INP, Institut Pascal, Université Clermont Auvergne, Clermont-Ferrand, France; ^2^ Centro Singular de Investigación en Tecnoloxías Intelixentes (CiTIUS), Universidade de Santiago de Compostela, Santiago de Compostela, Spain

**Keywords:** human-robot collaboration, decision-making, game theory, Nash equilibrium, interaction optimality

## Abstract

This paper proposes a new decision-making framework in the context of Human-Robot Collaboration (HRC). State-of-the-art techniques consider the HRC as an optimization problem in which the utility function, also called reward function, is defined to accomplish the task regardless of how well the interaction is performed. When the performance metrics are considered, they cannot be easily changed within the same framework. In contrast, our decision-making framework can easily handle the change of the performance metrics from one case scenario to another. Our method treats HRC as a constrained optimization problem where the utility function is split into two main parts. Firstly, a constraint defines how to accomplish the task. Secondly, a reward evaluates the performance of the collaboration, which is the only part that is modified when changing the performance metrics. It gives control over the way the interaction unfolds, and it also guarantees the adaptation of the robot actions to the human ones in real-time. In this paper, the decision-making process is based on Nash Equilibrium and perfect-information extensive form from game theory. It can deal with collaborative interactions considering different performance metrics such as optimizing the time to complete the task, considering the probability of human errors, etc. Simulations and a real experimental study on “an assembly task” -i.e., a game based on a construction kit-illustrate the effectiveness of the proposed framework.

## 1 Introduction

Nowadays, Human-Robot Collaboration (HRC) is a fast-growing sector in the robotics domain. HRC aims to make everyday human tasks easier. It is a challenging research field that interacts with many others: psychology, cognitive science, sociology, artificial intelligence, and computer science (Seel, 2012). HRC is based on the exchange of information between humans and robots sharing a common environment to achieve a task as teammates with a common goal (Ajoudani et al., 2018).

HRC applications can have social and/or physical benefits for humans (Bütepage and Kragic, 2017). Social collaboration tasks include social, emotional and cognitive aspects (Durantin et al., 2017) such as care for the elderly (Wagner-Hartl et al., 2020), therapy (Clabaugh et al., 2019), companionship (Hosseini et al., 2017), and education (Rosenberg-Kima et al., 2019). Social robots, such as Nao, Pepper, iCub, etc., are dedicated to this type of task; however, their physical abilities are very limited (Nocentini et al., 2019). For the physical HRC (pHRC), physical contacts are necessary to perform the task. They can occur directly between humans and robots or indirectly through the environment (Ajoudani et al., 2018). pHRC applications are mainly used in industrial environments [e.g., assembly, handling, surface polishing, welding, etc., (Maurtua et al., 2017)]. pHRC is also used in the Advanced Driver-Assistance Systems (ADAS) for autonomous cars (Flad et al., 2014).

Robots can adapt to humans in different situations by implementing five steps in a decision-making process (Negulescu, 2014): 1) gathering relevant information on possible actions, environment, and agents, 2) identifying alternatives, 3) weighing evidence, 4) choosing alternatives and selecting actions, and 5) examining the consequences of decisions. These steps are usually modeled in computer science using a decision-making method with a strategy and a utility function (Fülöp, 2005). The decision-making method models the whole situation (environment, actions, agents, task restrictions, etc.). The strategy defines the policy of choosing actions based on the value of their reward. The utility function (i.e., reward function) evaluates each action for each alternative by attributing a reward to it.

On the one hand, previous works, known as leader-follower systems (Bütepage and Kragic, 2017), focused the decision process on choosing the actions that increase the robot’s abilities to accomplish the task without considering how the collaboration is done. Such as in DelPreto and Rus (2019), where a human-robot collaborative team is lifting an object together, or in Kwon et al. (2019), where robots influence humans to change the pre-defined leader-follower agents to rescue more people when there is a plane or ship crash in the sea.

On the other hand, other works deal with maximizing the collaboration performance by promoting mutual adaptation (Nikolaidis et al., 2017b; Chen et al., 2020) or reconsidering the task allocation (Malik and Bilberg, 2019). However, they only consider one or two unchangeable performance metrics for this evaluation in their utility function: postural or ergonomic optimization (Sharkawy et al., 2020), time consumption (Weitschat and Aschemann, 2018), trajectory optimization (Fishman et al., 2019), cognitive aspects (Tanevska et al., 2020), and reduction of the number of human errors (Tabrez and Hayes, 2019).

In this paper, we optimize and quantitatively assess the collaboration between robots and humans based on the resulting impact of some changeable performance metrics on human agents. Hence, an optimized collaboration aims to bring a benefit to humans, such as getting the task done faster or reducing the effort of human agents. However, an unoptimized collaboration will bring nothing to humans or, on the contrary, will represent a nuisance, such as slowing them down or overloading them, even if the task is finally accomplished. The main contribution of this paper is that the proposed framework allows optimizing the performance, based on some changeable metrics, of the collaboration between one or more humans and one or more robots. Contrary to previous works, our framework allows us to easily change the performance metrics without changing the whole way the task is formalized since we isolate the impact of the metrics in the utility function.

The benefit of this contribution is to increase the collaboration performance without having to ameliorate the robot’s abilities. This is important in relevant practical cases: for instance, when using social robots that have great limitations (e.g., slowness in their movements and/or reduced dexterity), and it is not easy or even possible to ameliorate their abilities drastically. Therefore, our work provides an interesting solution to enhance collaboration performance with such limited robots.

Our framework uses the state-of-the-art decision-making process composed of: a decision-making method, a strategy, and a utility function. We divide the utility function into two main parts: the collaboration performance evaluated by a reward according to one or several performance metrics, and the task accomplishment, which is considered as a constraint since we only deal with achievable tasks.

**FIGURE 1:**
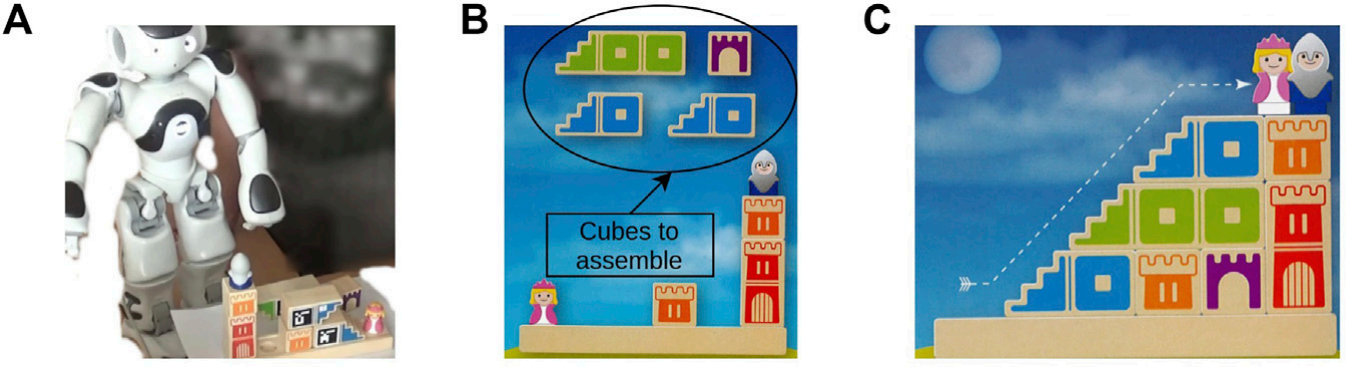
Agents solving the Camelot Jr. game. **(A)** Agents play sequentially: the human starts to play, and then it is the robot’s turn. **(B)** This puzzle starts with four cubes to assemble. **(C)** The cubes are correctly assembled, and the puzzle is solved (i.e., a path composed by cubes is created between both figurines).

The paper is organized as follows. First, we review related work in Section 2. Then, we present our framework formalization in Section 3. Section 4 includes all the details regarding the decision-making process. The effectiveness of our new formalization is shown in Section 5 based on simulated and experimental tests of an assembly task (i.e., a game^1^ that involves placing cubes to build a path between two figurines) shown in Figure 1. Finally, we sum up the effectiveness of our contribution and discuss the possible improvements in Section 6.

## 2 Related Work

In this section, we present the most popular methods, strategies, utility functions, and performance metrics used in the decision-making process of human-robot collaboration to place our contributions with regard to them. A decision-making method models the relationship between the agents, the actions, the environment, the task, etc. A strategy defines how to select the optimal actions each agent can choose based on the reward (utility) calculated by the utility function for each action. An optimal action profile is made up of the best actions each agent can choose. All methods and strategies can be used to perform different tasks, and there is no pre-written rule that implies that one will necessarily perform better than the others.

### 2.1 Decision-Making Methods

Decision-making methods are used, as mentioned before, to model the relationship between the task, the agents accomplishing it, their actions, and their impact on the environment. Probabilistic methods, deep learning, and game theory are considered among the most widespread decision-making methods.

Probabilistic methods are the first and most widely used in decision-making processes. Markov’s decision-making processes (e.g., Markov chains) are the most used ones. There are also studies based on other methods such as Gaussian processes (e.g., Gaussian mixtures), Bayesian processes (e.g., likelihood functions), and graph theory. In Roveda et al. (2021), a Hidden Markov Model (HMM) is used to teach the robot how to achieve the task based on human demonstrations, and an algorithm based on Bayesian Optimization (BO) is used to maximize task performance (i.e., avoid task failures while reducing the interaction force), and to enable the robot to compensate for task uncertainties.

The interest in using deep learning in decision-making methods began very early due to unsatisfactory results of probabilistic methods in managing uncertainties in complex tasks. In Oliff et al. (2020), Deep Q-Networks (DQN) are used to adapt robot behavior to human behavior changes during industrial tasks. The drawbacks of deep learning methods are the computation cost and the slowness of learning.

Game theory methods in decision-making processes have only recently been exploited. They can model most of the tasks performed by a group of agents (players) in collaboration or competition, whether the choice of actions is simultaneous [normal form also called matrix form modeling (Conitzer and Sandholm, 2006)] or sequential [extensive form also known as tree form modeling (Leyton-Brown and Shoham, 2008)]. The game theory methods have been used in different HRC applications, for instance, in analyzing and detecting the human behavior to adapt the robot’s one to it for reaching a better collaboration performance (Jarrassé et al., 2012; Li et al., 2019). Game theory has been also utilized in HRC in mutual adaptation to achieve industrial assembly scenarios (Gabler et al., 2017). We choose the game theory as a decision-making method due to its simplicity and effectiveness in modeling most interactions between a group of participants and their reactions to each other’s decisions. We specifically use the extensive form due to its sequential nature, which is suitable for HRC applications.

### 2.2 Decision-Making Strategies

The decision-making strategy is the policy of choosing actions based on the value of their reward calculated by the utility function (i.e., the reward function). We present the most used strategies for multi-criteria decision-making in HRC as well as some of their application areas. The following strategies are used intensively in deep learning and/or in Game theory (Conitzer and Sandholm, 2006; Leyton-Brown and Shoham, 2008):

• Dominance: All the actions whose rewards are dominated by others are eliminated. Researchers used it to assess the human’s confidence in a robot in Reinhardt et al. (2017).
• Pareto optimality: An action profile is Pareto optimal if we cannot change it without penalizing at least one agent. It is used, for example, in disassembly and remanufacturing tasks (Xu et al., 2020).
• Nash Equilibrium (NE): Each agent responds to the others in the best possible way. The best response is the best actions an agent can choose whatever others have done. This is the main strategy used in Game theory. In Bansal et al. (2020), a NE strategy is used to ensure human safety in a nearby environment during a pick-and-place task.
• Stackelberg duopoly model: The agents make their decision sequentially: one agent (the leader) makes their decision first, and all other agents (followers) decide after. The optimal action of the leader will be the one that maximizes its own reward and minimizes the follower’s rewards. This means that the leader has always the biggest reward. This strategy is used, for example, in a collaborative scenario between a human and a car to predict the driver’s behavior in this specific scenario (Li et al., 2017) such as the driver’s steering behavior in response to a collision avoidance control (Na and Cole, 2014).

### 2.3 Performance Metrics

After the decision-making process is settled and used to perform a task by a human-robot collaborative team, other works tend to evaluate the performance of the collaboration using some performance metrics. On the one hand, some works focused on evaluating one specific metric, as done in Hoffman (2019), where the author is evaluating several human-robot collaborative teams, performing different tasks, using the fluency metric. On the other hand, other works create a global framework to evaluate, in general, the HRC based on several metrics. In Gervasi et al. (2020), the authors developed a global framework to evaluate the HRC based on more than twenty performance metrics, among which the cognitive load and the physical ergonomics. Table 1 presents the main metrics considered, in the state-of-the-art, to evaluate the optimality of the collaboration. We present in the Supplementary Material a more detailed table that introduces more performance metrics and defines each metric according to its usage in different task types.

**TABLE 1:** Some metrics considered for the evaluation of HRC classified based on the task types (Steinfeld et al., 2006; Bütepage and Kragic, 2017; Nelles et al., 2018).

Task	Navigation	Perception	Management	Manipulation	Social	Common metrics that can be used for all task types
Performance metrics	Failure rate, accuracy, ergonomy or posture, time to completion, and rapidity	Velocity, accuracy, time to completion, effectiveness, and number of errors	Time delivery, time request, number of human and robot errors, trust, cognitive load	Positional accuracy and repeatability, velocity, dexterity, time to completion, and effort or force	Persuasiveness, engagement in social characteristics, trust, and compliance	Time to completion, number of human and robot errors, autonomy, cognitive load, and effectiveness

### 2.4 Utility Functions

The utility is a reward calculated by the utility function to express the value of an action. Thanks to these utilities, the decision-making strategy can choose the right actions. Some previous works in the literature only considered task accomplishment (and no performance metrics) in their utility functions because their focus was on complex task accomplishment. For example, in Nikolaidis et al. (2017a), a human-robot collaborative team was carrying a table to move it from one room to another. The goal was to ensure mutual adaptation between the agents by having the human also adapt to the robot. In this type of work, none of the performance metrics in Table 1 is considered.

More recent works include performance metrics (see Table 1). However, they considered that they are not changeable without significant changes in their framework. A relevant example is Liu et al. (2018) where, by changing the task allocation, the authors make the robot respect the real-time duration of the assembly process while following the necessary order to assemble the parts. In this case, they considered one metric (the time to completion) since respecting the part’s assembly order is a constraint to accomplish the task. However, this time metric cannot be replaced by another (e.g., effort or velocity) using this framework.

### 2.5 Contributions

Unlike the utility functions used in the state-of-the-art works, we take into account a changeable unrestricted number of performance metrics (from Table 1) that are usually optimized no matter how the human is behaving. To summarize our contributions, we propose a framework that allows us to:

• easily change the performance metrics from one scenario to another without changing anything in our formalization except the part in the utility function related to the metrics, and
• improve the collaboration performance without having to change the robot’s abilities.

In the following section, we define the problem formalization and present the utility function which optimizes the performance metrics and aims to accomplish the task as a constraint.

## 3 Formalization

A HRC^2^ consists of a global environment {**E**} and a task *T*. The environment state *E* ^*k*^ at each iteration *k* (with *k* ∈ [1, *k*_*f*_], where *k*_*f*_ is the final iteration of the task) comprises a group of *n* agents (humans and robots), each of them can carry out a finite set of actions (continuous or discrete). *E* ^*k*^ changes according to the actions chosen by the agents. The global environment {**E**} is the set of changes in the environment state at each iteration.

$$\left\{E\right\}=\left\{E^{1},E^{2},\ldots ,E^{k},\ldots ,E^{k_{f}}\right\}$$ (1)

Since the possible actions may change at each iteration, we define {**A**} as the global set of feasible actions for each iteration *k*: {**A**}^*k*^.

$$\left\{A\right\}=\left\{{\left\{A\right\}}^{1},{\left\{A\right\}}^{2},\ldots ,{\left\{A\right\}}^{k},\ldots ,{\left\{A\right\}}^{k_{f}}\right\}$$ (2)

The set {**A**}^*k*^ contains a set of feasible actions for each agent *i* (with *i* ∈ [1, *n*]) at iteration *k* denoted by ${\left\{A_{i}\right\}}^{k}$ .

$${\left\{A\right\}}^{k}=\left\{{\left\{A_{1}\right\}}^{k},\ldots ,{\left\{A_{i}\right\}}^{k},\ldots ,{\left\{A_{n}\right\}}^{k}\right\}$$ (3)

$A_{i,a}^{k}$ is the *a* ^*th*^ feasible action of agent *i* where *a* ∈ [1, *l*] and *l* is the number of feasible actions of the agent *i* at time *k*.

$${\left\{A_{i}\right\}}^{k}=\left\{A_{i,1}^{k},\ldots ,A_{i,a}^{k},\ldots ,A_{i,l}^{k}\right\}$$ (4)

At each iteration, an action profile ${\left(\vec{A}\right)}^{k}$ groups the actions chosen by each agent *i* denoted by $A_{i}^{k}\subset {\left\{A_{i}\right\}}^{k}$ .

$${\left(\vec{A}\right)}^{k}=\left(A_{1}^{k},\ldots ,A_{i}^{k},\ldots ,A_{n}^{k}\right)$$ (5)

**FIGURE 2:**
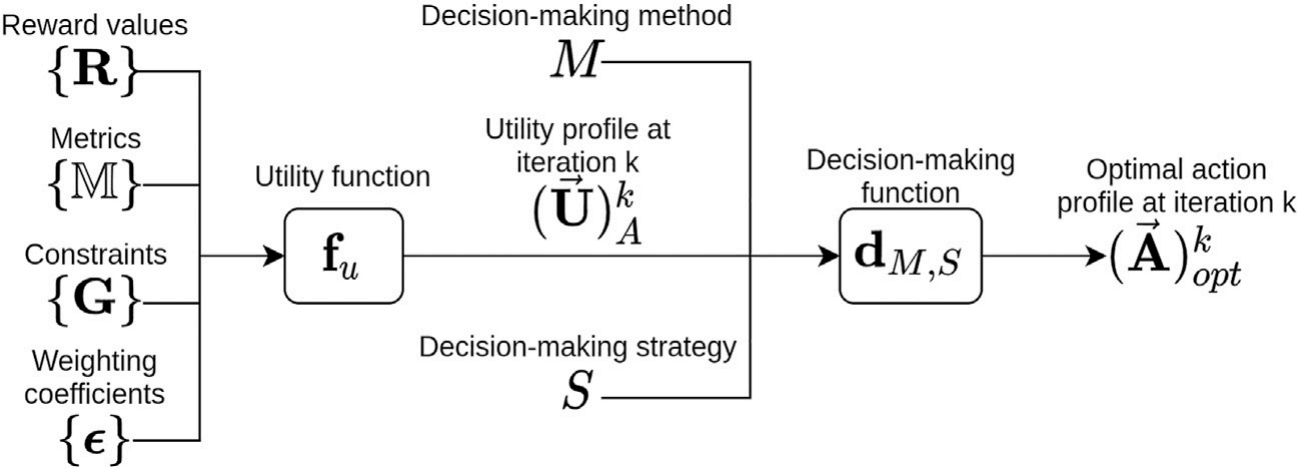
Block diagram of our formalization of the decision-making process used to calculate the optimal action profile ${\left(\vec{A}\right)}_{opt}^{k}$ at iteration *k*.

The optimal action profile ${\left(\vec{A}\right)}_{opt}^{k}$ at iteration *k* is computed through the decision-making function **d**_*M*,*S*_ as presented in Eq. 6. **d**_*M*,*S*_ relies on the decision-making method *M*, the decision-making strategy *S*, and the utility profile ${\left(\vec{U}\right)}_{A}^{k}$ that contains all the utilities for all possible actions {**A**}^*k*^ at iteration *k*. The decision-making method *M* takes into account the constraints related to the task such as the order in which the agents act, i.e., sequentially or simultaneously. The decision-making strategy *S* defines the way the agents must choose the actions according to their utilities contained in ${\left(\vec{U}\right)}_{A}^{k}$ , as presented in Figure 2.

$${\left(\vec{A}\right)}_{opt}^{k}=d_{M,S}\left({\left(\vec{U}\right)}_{A}^{k}\right)$$ (6)

The utility profile ${\left(\vec{U}\right)}_{A}^{k}$ is computed by the utility function **f**_*u*_ based on different sets including: 1) the set of performance metrics {M} (cf. Table 1), 2) the set of constraints {**G**} to be respected in order to make the task *T* progress for accomplishing it, 3) {**R**} the reward of each action in the profile action which is calculated according to the task and the metrics, and 4) {***ϵ***} a set of weighting coefficients (between 0 and 1) used to determine the importance of each metric (e.g., favoring one metric over the others, especially when it is in opposition to others). We get:

$${\left(\vec{U}\right)}_{A}^{k}=f_{u}\left(\left\{M\right\},\left\{G\right\},\left\{R\right\},\left\{\epsilon \right\}\right)=\left(U_{A_{1}}^{k},\ldots ,U_{A_{i}}^{k},\ldots ,U_{A_{n}}^{k}\right)$$ (7)

Let us discuss how one can make changes to the different elements involved in Eq. 7. To only change the scenario of the collaboration by changing the performance metrics {M} in **f**_*u*_, we first need to change the value of the metrics {M} , and the value of the reward {**R**} of each action, and afterwards recalculate the utilities ${\left(\vec{U}\right)}_{A}^{k}$ . To only modify the agent’s actions, the utilities ${\left(\vec{U}\right)}_{A}^{k}$ should be recalculated for the new actions. To change the task, we will need to modify the constraints {**G**} which define the task by setting the conditions that allow the agent to only choose among the actions which permit to make the task progress. It will then be necessary to recalculate the utilities ${\left(\vec{U}\right)}_{A}^{k}$ . We can, of course, combine several modifications (e.g., changing the performance metrics and the task) by making the appropriate adaptations (e.g., first modifying the metrics {M} , the rewards {**R**}, and the task constraints {**G**}, and afterwards recalculating the utilities ${\left(\vec{U}\right)}_{A}^{k}$ ).

To illustrate how Eqs 6, 7 can be settled, let us consider the example of a collaborative team composed of a human and a robot, each holding an edge of a gutter on which there is a ball (Ghadirzadeh et al., 2016). Their goal is to position the ball, for instance, in the center of the gutter. Our solution using our formalization for such task can be as follows:

•Agents: Agent 1 is the human, and agent 2 is the robot. Both agents are making decisions simultaneously.
•Human actions ${\left\{A_{1}\right\}}^{k}$ : They are the angles of inclination of the gutter. The actions are continuous. The set of human actions remains the same for all the iterations ( ${\left\{A_{1}\right\}}^{k}=\left\{A_{1}\right\}$ ).
•Robot actions ${\left\{A_{2}\right\}}^{k}$ : They are angles of inclination of the gutter by the robot end-effector. The decision-making method will provide the correspondent joint values needed to reach the desired position by the end-effector. The actions are continuous (since it is a continuous control task). The set of robot actions remains the same for all iterations ( ${\left\{A_{2}\right\}}^{k}=\left\{A_{2}\right\}$ ).
•Constraints {**G**}: The angles of inclination should be between [− 30°, 30°], other values will be penalized.
•Performance metrics {M} : Time to completion and human posture. Human posture is measured by ISO standards that define some uncomfortable work postures (Delleman and Dul, 2007). These uncomfortable postures (or positions) will lead, for example, to find that when the human inclines the gutter with an angle out of the interval [−20°, 20°], it is getting painful for them.
•Rewards {**R**}: They will be calculated by the following equation: −‖ *C*_*b*_ − *C*_*g*_ ‖ ∗ *λ*. Where *C*_*b*_ is the position of the center of the ball, *C*_*g*_ is the position of the center of the gutter (the desired position), and *λ* is a fixed gain for a case scenario. *λ* allows to privileged an action according to the performance metrics ( {M} ) and the constraints ({**G**}).
•Weighting coefficients {***ϵ***}: It is equal to 1 for both performance metrics.
•Decision-making method *M*: It is the reinforcement learning process that is based on trial and error learning. The agent 2 (the robot which learns) in state *s* makes an action *A*_2,*a*_ which changes the state to *s*′. The observation the agent got from the environment describes the changes that happened by moving from state *s* to *s*′. The reward (*R*(*s*, *A*_2,*a*_)) evaluates the taken action *A*_2,*a*_ (which leads to the new state *s*′) with respect to the desired learning goal. The state *s* is made up of *C*_*b*_, *C*_*g*_, and the position of the robot’s end-effector. The learning procedure of all reinforcement learning algorithms consists of learning the value that is attributed to the state *V*(*s*) defined below.
•Decision-making strategy *S*: It is the dominance strategy. Once the *V*(*s*) are learned for all possible states, the optimal actions can be chosen. Most of the reinforcement learning algorithms are based on the Bellman equation for choosing the optimal actions (Nachum et al., 2017):

$$V\left(s\right)=\underset{A_{2,a}}{\max}\left(R\left(s,A_{2,a}\right)+\gamma V\left(s^{\prime }\right)\right)$$ (8)

*γ* is the discount factor that determines how much agent 2 cares about rewards in the distant future relative to those in the immediate future. $\underset{A_{2,a}}{\max}$ is the strategy *S* for choosing the action (i.e., dominance strategy).

The decision-making method manages the way agents act (simultaneously or sequentially) as well as the different types of actions (continuous or discrete). It is also necessary to ensure that the decision-making strategy can handle the nature of the actions (discrete or continuous) and how they are chosen (sequentially or simultaneously). As our framework allows us to easily change the decision-making method and strategy, we just have to select them according to the nature of the actions and how they are chosen. Figure 2 summarizes our formalization of the decision-making process using a block diagram. In Section 4, we explain the selected decision-making method and strategy in our experiments as well as the performance metrics that can be taken into consideration.

## 4 Approach

To illustrate our contributions, we define a constant decision-making method *M* and strategy *S*. We assume as decision-making method the Perfect-Information Extensive Form (PIEF) of the game theory (environment and actions are known) in which the full flow of the game is displayed in the form of a tree. Using Nash Equilibrium as the strategy of the decision-making process ensures optimality regarding the choice of the actions, which is what we seek to guarantee.

### 4.1 Perfect-Information Extensive Form

As decision-making method *M* in Eq. 6 we used the Perfect-Information Extensive Form (PIEF). Using this method, the agent has all the information about the actions and decisions of other agents and the environment. A game (or task or application) in PIEF in game theory is represented mathematically by the tuple $T=\left(\left\{N\right\},\left\{A\right\},\left\{H\right\},\left\{Z\right\},\chi ,\rho ,\sigma ,\left\{\vec{U}\right\}\right)$ (Leyton-Brown and Shoham, 2008), with:

• *T* represents the game (i.e., the task) as a tree (graph) structure.
• {**N**} is a set of *n* agents.
• {**A**} is a set of actions of all agents for all iterations.
• {**H**} is a set of non-terminal choice nodes. A non-terminal choice node represents an agent that chooses the actions to perform.
• {**Z**} is a set of terminal choice nodes; disjoint from {**H**}. A terminal choice node represents the utility values attributed to the actions $A_{i}^{k}$ each agent *i* chose in an alternative (i.e., a branch of the tree).
• ***χ***: {**H**}↦{**A**}_*@H*_ is the action function, which assigns to each choice node *H* a set of possible actions {**A**}_*@H*_.
• ***ρ***: {**H**}↦{**N**} is the agent function, which assigns to each non-terminal choice node an agent *i* ∈ {**N**} who chooses an action in that node.
• ***σ***: {**H**} × {**A**}↦{**H**} ∪ {**Z**} is the successor function, which maps a choice node and an action to a new choice node or terminal node.
• $\left\{\vec{U}\right\}=\left\{{\left(\vec{U}\right)}_{A}^{1},\ldots ,{\left(\vec{U}\right)}_{A}^{k},\ldots ,{\left(\vec{U}\right)}_{A}^{k_{f}}\right\}$ is the global utility profile for all iterations.

We apply this structure to represent the task in the following sections. In our case, since the number of nodes is small, ***χ***, ***ρ***, and ***σ*** are straightforward functions (cf. Figure 4).

From a high-level perspective, a perfect-information game in extensive form is simply a tree (e.g., Figure 4) which consists of:

• Non-terminal nodes (squares): each square represents an agent that will choose actions.
• Arrows: each one represents a possible action (there are as many arrows as available actions ${\left\{A_{i}\right\}}^{k}$ for agent *i* at iteration *k*).
• Terminal nodes (ellipses): each ellipse represents the utilities calculated for each action chosen by each agent in an alternative (i.e., a branch of the tree).

Note that this kind of tree is made for all the possible alternatives (considering all the actions an agent might choose) even if some of them will never happen (the agent will never choose some of the available actions). In this way, the tree represents all possible reactions of each agent to any alternative chosen by the others, even if, in the end, only one of these alternatives will really happen.

### 4.2 Subgame Perfect Nash Equilibrium

As decision-making method *S* in Eq. 6 we used Nash Equilibrium (NE). The game *T* can be divided into subgames *T* ^*k*^ at each iteration. In game theory (Leyton-Brown and Shoham, 2008), we consider a subgame of *T* (in PIEF game) rooted at node *H* as the restriction of *T* to the descendants of *H*. A Subgame Perfect Nash Equilibrium (SPNE) of *T* is all action profiles ${\left(\vec{A}\right)}^{k}$ such that for any subgame *T* ^*k*^ of *T*, the restriction of ${\left(\vec{A}\right)}^{k}$ to *T* ^*k*^ is a Nash Equilibrium of *T* ^*k*^.

Nash Equilibrium in pure strategy (game theory) at iteration *k* is reached when each agent *i* best responds to the others (denoted by − *i*). The Best Response (BR) at *k* is defined mathematically as:

$$A_{i}^{*k}\in BR\left(A_{-i}^{k}\right)\text{ iff }\forall A_{i}^{k}\in \left\{A_{i}^{k}\right\},U_{A_{i}^{*}|A_{-i}}^{k}\geq U_{A_{i}|A_{-i}}^{k}$$ (9)

Hence, NE will ultimately be expressed as follows: ${\left(\vec{A}\right)}_{opt}^{k}=\left(A_{1}^{k},\ldots ,A_{i}^{k},\ldots ,A_{n}^{k}\right)$ is an optimal profile of actions following Nash’s equilibrium in pure strategy iff $\forall i,A_{i}^{k}\in BR\left(A_{-i}^{k}\right)$ .

From a high-level perspective, to ensure that the actions chosen by one agent are following the NE strategy, it is enough to verify that each agent chooses the actions that have the maximum possible utilities.

### 4.3 Performance Metrics

As long as a metric can be formulated mathematically or at least can be measured during the execution of the task and expressed as a condition to calculate the task reward, it can be considered in choosing the actions through the performance metrics {M} (some examples are given in Table 1). In the next section, we present the tests conducted, and we mention the chosen performance metrics for each scenario.

## 5 Experiments

We conduct real and simulated tests to prove the effectiveness of our formalization. We test three different utility function case scenarios in which the reward values change according to the chosen performance metrics. In the state-of-the-art case scenario, no metric is optimized. In the real experimental tests, the time to completion metric is optimized. In the simulated tests, we optimize the time to completion by considering the probability of human errors and the time each agent takes to make an action.

### 5.1 The Task

We chose to solve Camelot Jr. as a task. To successfully complete this task, all the cubes must be positioned correctly to build a path between the two figures. We have divided the task completion process into iterations during which each agent chooses an action sequentially.

#### 5.1.1 Experiments Context

We make the collaborative team ({**N**}), composed of a human (*h*) and the humanoid robot Nao (*r*), do a task (*T*) that consists of building puzzles (cf. Figure 1). Nao is much slower than the human ( $t_{A_{r}}>t_{A_{h}}$ ) in doing physical tasks (e.g., pick-and-place tasks), and we want to minimize the total task time ( {M} ). This slowness depends on the nature of the robot itself (its motor capacity combined with the use of its camera) and the complexity of the puzzle. For the robot, the puzzle is more complex as the number of cubes to assemble increases. It is quite different for the human the complexity depends on their “intelligence” which means that the puzzle is easier as the human is “intelligent”. By “intelligent”, we mean that the human can discover rapidly and without making mistakes where the correct position of each cube is.

The advantage of collaborating with the robot is that it knows the solution to the construction task. Therefore, the robot is always performing well, even if it is slower than the human. The human agent, however, can make mistakes. The human begins to play, and then, it is the robot’s turn. The robot will correct the human’s move if this move is wrong. The changes in the robot’s decision-making between the three case scenarios, including all the details we will present in the following sections, are shown in Figure 6. The implementation procedure and computation times for the conducted experiments are presented in the Supplementary Material.

#### 5.1.2 Assumptions

To illustrate the contributions of this paper, we consider the following assumptions:

• The task is always achievable. We solve the task while optimizing the performance metrics through the utility function. The optimization of the metrics does not have an impact on the solvability of the task.
• We limit the number of agents to two: a human (*h*) and a robot (*r*). Hence, {**N**} = {*h*, *r*} ⇒ *n* = 2.
• We limit agents to choose only one discrete action per iteration (i.e., $|A_{i}^{k}|=1$ ) and to maximize only one metric (time to completion) in the real experiment and two metrics (time to completion by considering the probability of human errors) in the simulated experiments.
• The task is performed sequentially through iterations. An iteration *k* includes the human making an action, then the robot reacting.
• The agent set of actions and the time the agent takes to make an action are invariable by iteration.

#### 5.1.3 The Actions

The set of human actions Eq. 10 and the set of robot actions Eq. 11 are the same at every iteration, and each one of them consists of three actions:

• *A*_*h*,*g*_ ≡ *A*_*r*,*g*_: perform the good action (i.e., grasp a free cube and release it at the right place).
• *A*_*h*,*w*_ ≡ *A*_*r*,*w*_: wait (i.e., the agent does nothing and passes its turn).
• *A*_*h*,*b*_: perform the bad action (i.e., the human makes an error: grasping a free cube and releasing it at the wrong place).
• *A*_*r*,*c*_: correct a bad action (i.e., the robot removes the cube from the wrong place).

$${\left\{A_{h}\right\}}^{k}=\left\{A_{h}\right\}=\left\{A_{h,g},A_{h,w},A_{h,b}\right\}$$ (10)

$${\left\{A_{r}\right\}}^{k}=\left\{A_{r}\right\}=\left\{A_{r,g},A_{r,w},A_{r,c}\right\}$$ (11)

#### 5.1.4 Utility Calculation

The following equation is the adaptation of Eq. 7 to the current task. So, the utility of every available action *a* for each agent *i* is calculated as follows:

$$U_{A_{i,a}}^{k}=U_{A_{i,a}}=\left(\frac{1}{t_{A_{i,a}}}\times G_{A_{i,a}}\times R_{A_{i,a}}\right)t$$ (12)

with:

• $t_{A_{i,a}}$ : the duration of action *a* of agent *i*,
• *t*: the total time for an iteration ( $t=\sum_{i=1}^{n}t_{A_{i,a}}$ , here having *n* = 2, therefore $t=t_{A_{h,a}}+t_{A_{r,a^{\prime }}}$ ),
• $G_{A_{i,a}}$ : the constraint that ensures the task progression by penalizing the actions which make the task regress (cf. Table 2),
• and $R_{A_{i,a}}$ : the reward of action *a* of agent *i*.

**TABLE 2:** The value of the constraint of the task accomplishment for each action: making the task progress ( $G_{A_{i,a}}=1$ ), making no progression ( $G_{A_{i,a}}=0$ ), and making the task regress ( $G_{A_{i,a}}=-1$ ).

	Action	$G_{A_{i,a}}$	Task progress
Human	*A*_*h*,*g*_	1	Progression
	*A*_*h*,*w*_	0	No progression
	*A*_*h*,*b*_	− 1	Regression
Robot	*A*_*r*,*g*_	1 if *A*_*h*_ ≠ *A*_*h*,*b*_ or − 1 otherwise	Progression
	*A*_*r*,*w*_	0	No progression
	*A*_*r*,*c*_	1 if *A*_*h*_ = *A*_*h*,*b*_ or − 1 otherwise	Progression

#### 5.1.5 Strategy of Action’s Choice

In our formalization, we mentioned that the agents are choosing Nash Equilibrium (NE) as the decision-making strategy. But since the behavior (the decision-making strategy) of each human is different from one to another, we cannot claim that they will follow the NE for choosing their actions. For the robot, however, we restrict it to choose the actions by using the NE strategy. That is why the robot is choosing the action with the highest utility knowing the one chosen by the human. Note that, in our case scenarios, the robot reacts to the human’s action since they are doing the task sequentially, and the human starts.

### 5.2 State-of-the-Art Utility Function

In state-of-the-art techniques, there is no optimization of the task. This is equivalent to always consider: $\forall a,i\;R_{A_{i,a}}=1$ in our approach (in Eq. 12). For each iteration (each agent chooses an action with a utility), we can represent the task with the tree structure of Figure 4A. We will refer in the rest of the article to this case scenario by using *C*_1_.

In this case, using NE, the robot’s reaction to the human action will be as follows: *A*_*r*,*g*_ if the human chose *A*_*h*,*g*_ or *A*_*h*,*w*_, and *A*_*r*,*c*_ if the human chose *A*_*h*,*b*_.

### 5.3 Real Experiments with Time Metric

We conducted tests^3^ with a group of 20 volunteers. The objectives were to prove that the framework is applicable for a real task and to check human adaptation to the robot.

#### 5.3.1 Experiment Procedure

After explaining the game rules to the participants, we asked them to complete two puzzles to make sure they understood the gameplay. Afterward, we asked each participant to complete three puzzles, chosen randomly among five, by collaborating with the Nao Robot.

**FIGURE 3:**
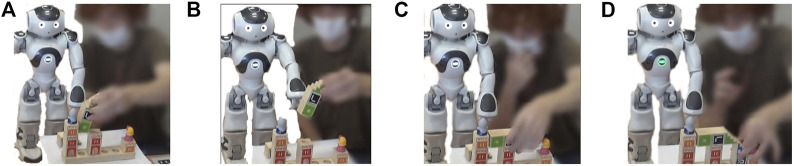
Example of the solving steps of the puzzle two by a participant and Nao. **(A)** The human puts a cube in a wrong position. **(B)** Nao asks him to remove that cube. **(C)** The human puts a cube in a correct position, then the robot does nothing. **(D)** The human puts another cube in a correct position and the puzzle is solved.

The participant began the game. Then, it was Nao’s turn. It continued until the puzzle was done. At each time, the participant had 20 s ( $t_{A_{h}}$ ) to make an action or to decide to skip their turn. Nao takes on average 60 s $\left(t_{A_{r}}\right)$ to do an action. It was skipping its turn when humans were well-doing and correcting them when they made an error. Nao did not move the cubes on its own (for human safety), but it was showing and telling the human which cube should be moved and where by pointing it. Figure 3 illustrates, as an example, the steps of solving puzzle two by a participant and Nao.

#### 5.3.2 Utility Function for Optimizing the Time

The reward values Eq. 13 in the utility function Eq. 12 ensure to maximize the time metric by penalising the action taken by the robot (the slower agent, i.e., $R_{A_{i,a}}=-1$ ) if the human (the faster agent denoted by *i*′) chooses the correct action (denoted by *a*′). This penalization will prevent the robot from interfering with the human actions if the human makes the right decision:

$$R_{A_{i,a}}=\left\{\begin{array}{cc} -1\; & \text{if}\;G_{A_{i,a}}>0\text{ and }G_{A_{i^{\prime },a^{\prime }}}=1\text{ and }t_{A_{i^{\prime },a^{\prime }}}<t_{A_{i,a}}\\ 1\; & \text{otherwise} \end{array}\right.$$ (13)

**FIGURE 4:**
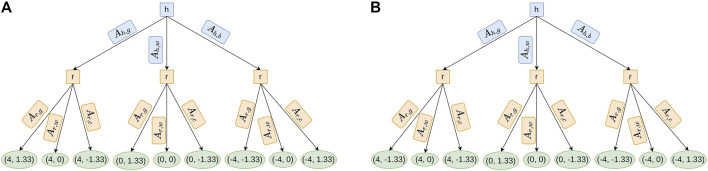
Tree representation of the task based on the utility function in *C*_1_ and *C*_2_. Notice that the difference between both figures is the utility value of the action *A*_*r*,*g*_, of the robot (1.33 and −1.33). It is because *C*_1_ (on the contrary of *C*_2_) does not minimize the time, so the robot continues to make an action even if the robot is slower than the well-performing human. **(A)** This tree is obtained by simulating an iteration of the task without optimization (*C*_1_). The utilities (first for human agent and second for robot in green ellipses) are calculated for $t_{A_{h}}=20\;s,t_{A_{r}}=60\;s$ and *t* = 80 s. **(B)** This tree is obtained by simulating an iteration of the task optimized by the time metric (*C*_2_). The utilities (first for human agent and second for robot in green ellipses) are calculated for $t_{A_{h}}=20\;s,t_{A_{r}}=60\;s$ and *t* = 80 s.

Thus, for each iteration, we can represent the task with the tree structure of Figure 4B. We will refer in the rest of the article to this case scenario using *C*_2_. In this case, using NE, the robot’s reaction to the human action will be as follows: *A*_*r*,*w*_ if the human chose *A*_*h*,*g*_, *A*_*r*,*g*_ if the human chose *A*_*h*,*w*_, and *A*_*r*,*c*_ if the human chose *A*_*h*,*b*_.

#### 5.3.3 Results

Experiments with humans (presented in Section 5.3.1) were those where the robot used the utility function optimizing the time metric (case 2 (*C*_2_)). It was very difficult to have enough participants to also test the case where the robot does not optimize any metric (the state-of-the-art case (*C*_1_)). The only change in the procedure of the experiments using *C*_1_ will be that even if the human is well-doing, the robot will not pass its turn (*A*_*r*,*w*_) but will perform the good action (*A*_*r*,*g*_). Hence, to compare the achieved results of our technique and the state-of-the-art techniques, we assumed that human actions remain the same in the case *C*_1_ as in the case *C*_2_, and we merely changed the robot reactions.

**FIGURE 5:**
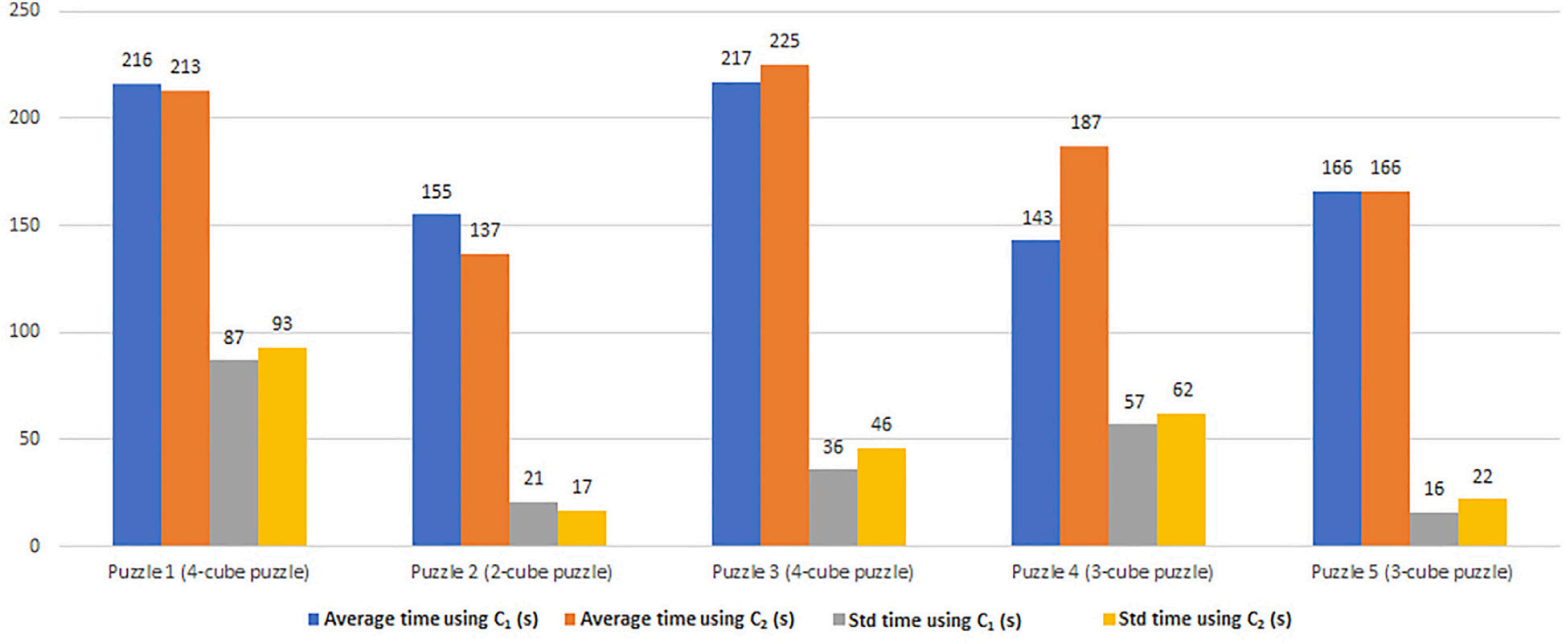
The average time and the standard deviation in seconds of the time taken to do the task with the state-of-the-art utility function (*C*_1_) and the utility function used to optimize the time (*C*_2_), which is our contribution.

We chose to keep human actions unchanged between the two cases to ensure that only the switching of the utility function (*C*_2_ to *C*_1_) affects the robot reaction and not the influence of human behavior. Table 3 provides an example of a scenario for solving puzzle two with *C*_2_ and *C*_1_ (Figure 3). We also calculated in Figure 5 the average time and the standard deviation of the measured times among the experiments (*C*_2_) and the deducted times (*C*_1_).

**TABLE 3:** The adaptation of time calculation from *C*_2_ to *C*_1_ for the resolution of one scenario of puzzle two.

		Iteration 1	Iteration 2	Iteration 3	Total time (s)
*C*_1_	Human actions	*A*_*h*,*b*_ (20s)	*A*_*h*,*g*_ (20s)		160
	Robot reactions	*A*_*r*,*c*_ (60s)	*A*_*r*,*g*_ (60s)		
	Iteration time	80 s	80 s		
*C*_2_	Human actions	*A*_*h*,*b*_ (20s)	*A*_*h*,*g*_ (20s)	*A*_*h*,*g*_ (20s)	140
	Robot reactions	*A*_*r*,*c*_ (60s)	*A*_*r*,*w*_ (20s)		
	Iteration time	80s	40s	20s	

In *C*_2_, we assumed that if the human does the good action once, they will continue to do it each time. We notice from Figure 5 that *C*_1_ works better when the human is not “intelligent”, i.e., they make lots of errors. That is why, the standard deviation values using *C*_2_ are bigger than using *C*_1_. This is the case for the last three puzzles where the average time using *C*_2_ is bigger than using *C*_1_. For the first puzzle, however, the average time using *C*_2_ is smaller than using *C*_1_, but the standard deviation values using *C*_2_ are bigger than using *C*_1_. The standard deviation values of this puzzle (using *C*_1_ and *C*_2_) are the biggest ones among all puzzles presented in Figure 5. Having big standard deviation values means that this puzzle was harder to solve for some participants and easier for others. That is why the average time and the standard deviation values using *C*_2_ and *C*_1_ do not have the same trend.

On the contrary, *C*_2_ performs better when the human is “intelligent”. Therefore, the time taken to accomplish the task depends on human “intelligence” that is related to the probability of human errors and the ratio between the time each agent takes to do an action. Without taking into account these two additional metrics, we cannot optimally ensure to minimize the time to completion when the human makes many mistakes.

In the next case (*C*_3_), we present a third utility function that takes into account the time taken by the agents to make an action and optimizes the time to completion by encouraging the human agent to reduce the number of errors. Each metric has the same weight *ϵ* = 1 (Eq. 7) since all these metrics are compatible. It means that optimizing one metric depends on optimizing the others.

### 5.4 Simulated Experiments with Time and Number of Human Errors Metrics

**FIGURE 6:**
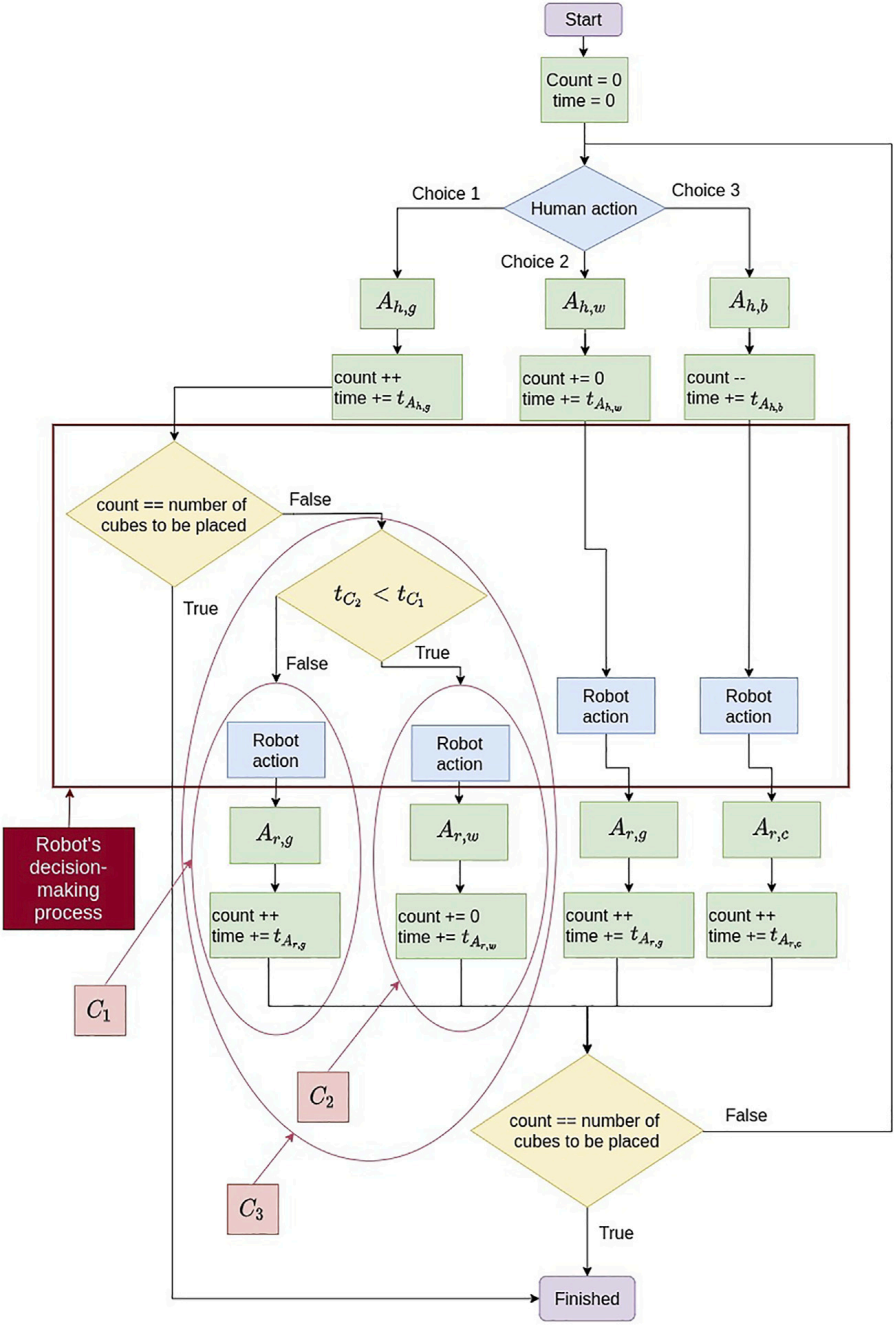
*C*_3_ algorithm block diagram.

We use case (*C*_3_) to prove that our framework can handle the changes in the performance metrics from one case scenario to another. In this case (*C*_3_), we select between *C*_1_ and *C*_2_ the case that minimizes the total time by considering the probability of human errors and the ratio between the time each agent takes to make an action. The difference between *C*_1_ and *C*_2_ lies in the robot reaction when the human agent makes the good action (*A*_*h*,*g*_). With *C*_1_, the robot makes the good action (*A*_*r*,*g*_), while with *C*_2_, the robot decides to wait (*A*_*r*,*w*_), to not slow down the human. Figure 6 presents an algorithmic block diagram showing which case the robot will choose to make an action.

#### 5.4.1 Assumptions on Humans

We did not have enough participants to do real tests so we chose to do simulated tests. For this, we simulated the human decision process as a probability distribution among the set of feasible actions such that: *P*(*A*_*h*,*g*_) = *I*_1_, *P*(*A*_*h*,*w*_) = *I*_2_, and *P*(*A*_*h*,*b*_) = *I*_3_ = 1 − (*I*_1_ + *I*_2_). *I*_1_, *I*_2_, and *I*_3_ are variable from one participant to another and 0 < *I*_1_ + *I*_2_ ≤ 1.

#### 5.4.2 Utility Function for Optimizing the Time to Completion While Considering the Probability of Human Errors

Compares to Eq. 12, only the reward values ( $R_{A_{i,a}}$ ) change. The reward values of the utility function for *C*_3_ are calculated by the following function:

$$R_{A_{i,a}}=\left\{\begin{array}{cc} -1 & \text{if}\;G_{A_{i,a}}>0\text{ and }G_{A_{i^{\prime },a^{\prime }}}=1\text{ and }t_{C_{2}}<t_{C_{1}}\\ 1 & \text{otherwise} \end{array}\right.$$ (14)

Where $t_{C_{2}}<t_{C_{1}}$ decides which case (1 or 2) is the best to optimize the total time (cf. Figure 6) and thus reduce the number of human errors. So, if $t_{C_{2}}<t_{C_{1}}$ is true, *C*_2_ will be faster than *C*_1_, and vice versa. *t*_*C*_ is the generic equation for calculating time payoffs $t_{C_{1}}$ and $t_{C_{2}}$ Eq. 15. It considers the probability that the human agent will perform each feasible action (*P*(*A*_*i*′_) = probability distribution of human actions) which we assume as known and the time that the agents will take to make an action. $t_{A_{i^{\prime },a^{\prime }}}$ is the time required for the other agent *i*′ (i.e., human) to make the chosen action *a*′ and $t_{A_{i,a}}$ is the time taken by the agent *i* (i.e., robot) to react by making the action *a*. *N*_*c*_ is the number of cubes correctly placed by taking actions *a* and *a*′.

*C*_2_ did not work well because it was assuming that if the human does the good action once, they will continue to do it each time. That is why in Eq. 13 the comparison of the times ( $t_{A_{i^{\prime },a^{\prime }}}<t_{A_{i,a}}$ ) was not including the probability of the human actions (including probability of making errors). In case the human often performs the bad action (e.g., *I*_3_ ≥ 0.6), the robot is encouraged not to wait but to perform the good action (*C*_1_), despite its slowness. This is done to reduce the number of iterations and thus reduce the number of times the human will make a mistake, as they will have fewer turns to play (i.e., reducing the number of human errors). That is why in *C*_3_, we consider the probability distribution of human actions, including that of doing the bad action (committing errors) while calculating *t*_*c*_ (cf. Eq. 14). The robot chooses *C*_1_ if the human will make many errors and *C*_2_ in the opposite case.

$$t_{C}=\frac{\overset{l}{\underset{a^{\prime }=1}{\sum }}P\left(A_{i^{\prime },a^{\prime }}\right)\left(t_{A_{i^{\prime },a^{\prime }}}+t_{A_{i,a}}\right)}{\overset{l}{\underset{a^{\prime }=1}{\sum }}P\left(A_{i^{\prime },a^{\prime }}\right)N_{c}}$$ (15)

#### 5.4.3 Simulation Conditions

A simulated test depends on:

• The values of *I*_1_ and *I*_2_ (we tested for *I*_1_ = (0 : 0.1 : 1) and *I*_2_ = (0 : 0.1 : 1) except for *I*_1_ = *I*_2_ = 0).
• The ratio between $t_{A_{h}}$ and $t_{A_{r}}$ (we tested for 1/1, 1/1.5, 1/2, 1/3, 1/4, 1/5).
• The number of cubes required to solve the puzzle (we tested for 2, 3, 4, and 5).
• The number of simulations (10000) we conducted to calculate the average time and the standard deviation.

#### 5.4.4 Simulation Results

We illustrate the efficiency of our utility function *C*_3_ by showing the improvement in time to completion and the reduction of the number of human errors obtained while solving the puzzles.

##### 5.4.4.1 Time Improvement

We validate the efficiency of our utility function *C*_3_ by comparing the resulted average total times with similar cases using *C*_1_ over 10000 simulations^4^. Like real experiments, we assumed that human actions are constant, and we change merely the robot actions.

**FIGURE 7:**
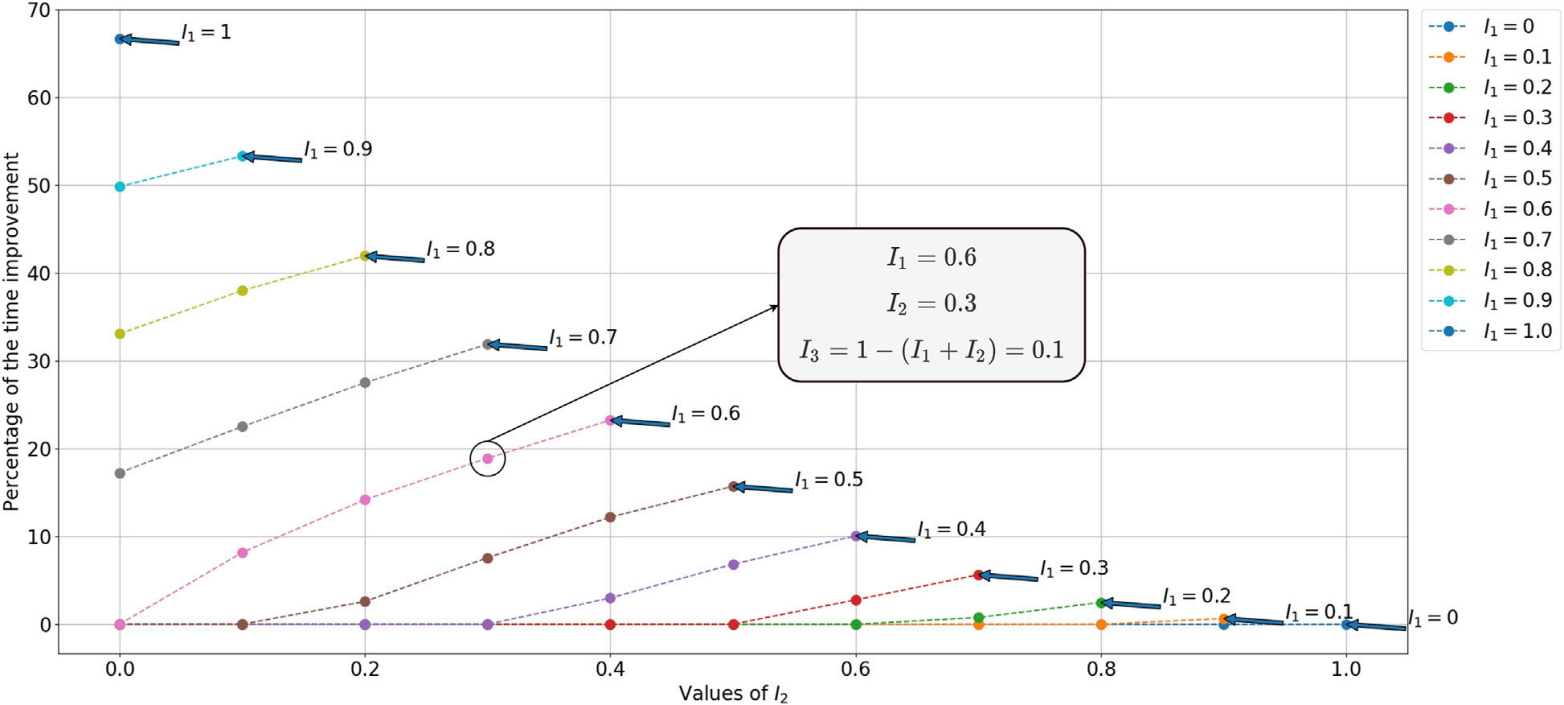
Percentage of time improvement between *C*_3_ and *C*_1_ for a 4-cube puzzle. $t_{A_{h}}=\left\{15,0,15\right\}$ and $t_{A_{r}}=\left\{75,0,75\right\}$ , so the ratio $t_{A_{h}}/t_{A_{r}}=1/5$ . *P*(*A*_*h*,*g*_) = *I*_1_, *P*(*A*_*h*,*w*_) = *I*_2_, and *P*(*A*_*h*,*b*_) = *I*_3_ = 1 − (*I*_1_ + *I*_2_). In this figure, each dotted line is equivalent to a specific *I*_1_ value. Each dot corresponds to a *I*_2_ value (read on the x-axis). For each dot knowing *I*_1_ and *I*_2_, we can deduce its *I*_3_ value using *I*_3_ = 1 − (*I*_1_ + *I*_2_). For illustrating, we give *I*_1_, *I*_2_, and *I*_3_ values of the dot marked in the figure.

We calculate the time improvement Eq. 16 by comparing the average total times $\left({\bar{D}}_{C_{3}}\right)$ calculated using the utility function of *C*_3_ to the average total times $\left({\bar{D}}_{C_{1}}\right)$ calculated using the state-of-the-art utility function (i.e., *C*_1_). This is illustrated in Figure 7 for a 4-cube puzzle with a ratio $t_{A_{h}}/t_{A_{r}}=1/5$ . As it can be observed, the experiment times are improved up to 66.7*%*. Another example is given in Supplementary Material for a 3-cube puzzle with a ratio $t_{A_{h}}/t_{A_{r}}=1/3$ . The percentage of the time improvement depends on how much the human participant is “intelligent”.

$$\text{Percentage of time improvement}=\frac{{\bar{D}}_{C_{1}}-{\bar{D}}_{C_{3}}}{{\bar{D}}_{C_{1}}}\;*\;100$$ (16)

Theoretically, however, this percentage can reach a value close to 100*%* for a very small time taken by the human (which lead to a very small ${\bar{D}}_{C_{3}}$ ) and a very big time taken by the robot (which lead to a very big ${\bar{D}}_{C_{1}}$ ). We can note that having the time improvement percentage equal to 0 signifies that we are using *C*_1_; while utilizing *C*_2_ increases the value of the time improvement percentage. It means that in the worst-case scenario, the efficiency of our method is as the state-of-the-art peers.

##### 5.4.4.2 Reduction of the Number of Human Errors

For reducing the time to completion, we consider the probability of human errors in Eq. 15. So, we choose between *C*_1_ and *C*_2_ the case which minimizes the time by reducing the number of iterations needed for solving the puzzle. This means choosing the case which reduces the number of human errors as explained in Section 5.4.2. We calculate, in Eq. 17, the percentage of human errors reduction (PHER) using the difference between the predicted probability of human errors *I*_3_ and the average (over the 10000 simulations) measured probability of human errors $\bar{\frac{N_{h_{e}}}{N_{h_{a}}}}$ .

$$\text{Percentage of human errors reduction}=\left\{\begin{array}{cc} \left(\frac{I_{3}-\left(\;\bar{\frac{N_{h_{e}}}{N_{h_{a}}}}\;\right)}{I_{3}}\right)\;*\;100 & \text{if}\;I_{3}>0\\ 0 & \text{if }I_{3}=0 \end{array}\right.$$ (17)

Where *I*_3_ is the predicted probability that the human makes a wrong move (makes an error), $N_{h_{e}}$ the measured number of human errors, and $N_{h_{a}}$ the measured total number of human actions. So, $\frac{N_{h_{e}}}{N_{h_{a}}}$ will be the measured probability that the human makes an error after one simulation. The reduction of the number of human errors is as big as $\bar{\frac{N_{h_{e}}}{N_{h_{a}}}}$ is small.

**FIGURE 8:**
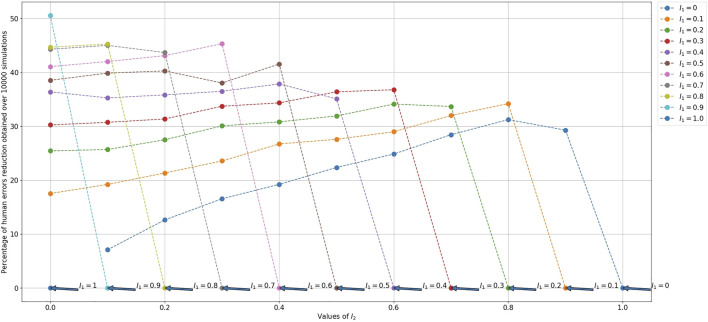
Percentage of human errors reduction between the predicted probability of human errors and the measured one for a 2-cube puzzle. $t_{A_{h}}=\left\{15,0,15\right\}$ and $t_{A_{r}}=\left\{15,0,15\right\}$ , so the ratio $t_{A_{h}}/t_{A_{r}}=1/1$ . *P*(*A*_*h*,*g*_) = *I*_1_, *P*(*A*_*h*,*w*_) = *I*_2_, and *P*(*A*_*h*,*b*_) = *I*_3_ = 1 − (*I*_1_ + *I*_2_). In this figure, each dotted line is equivalent to a specific *I*_1_ value. Each dot corresponds to a *I*_2_ value (read on the x-axis). For each dot knowing *I*_1_ and *I*_2_, we can deduce its *I*_3_ value using *I*_3_ = 1 − (*I*_1_ + *I*_2_).

The reduction percentage of the number of human errors increases with the reduction of $t_{A_{r}}$ (the time the robot takes to make an action) and the reduction of the number of cubes that should be assembled to solve a puzzle. In other words, the human will have fewer turns to play and so fewer chances to make mistakes. The best result we got is presented in Figure 8 (for a 2-cube puzzle with $t_{A_{h}}=t_{A_{r}}$ ): the reduction percentage of the number of human errors is up to 50.6*%*. The result can be better in case that the robot is faster than the human in performing an action ( $t_{A_{r}}<t_{A_{h}}$ ). Note that, when the *I*_3_ is equal to 0*%*, the percentage of human errors reduction is also equal to 0*%*. It means that the human never makes errors so, there is nothing that needs to be improved. Another example is presented in Supplementary Material for a 3-cube puzzle with a ratio $t_{A_{h}}/t_{A_{r}}=1/3$ .

## 6 Conclusion and Future Work

We propose a new formalization of the decision-making process to perform the task and accomplish it more efficiently. We assess through the experiments that our formalization can be applied to feasible tasks and optimize the human-robot collaboration in terms of all defined metrics. We also prove through the experiments that we can change the three studied case scenarios by changing the performance metrics in the utility function (i.e., reward function) without changing the entire framework.

Validating this, experiments are carried out by simulating the task of solving the construction puzzle. It shows that using our proposed utility function instead of the state-of-the-art utility function improves the experiment time up to 66.7*%*, hence improves the human-robot collaboration without extending the robot’s abilities. Theoretically, this improvement can reach a value close to 100*%*. We also got a percentage of human errors reduction up to 50.6*%* by considering the predicted probability that the human makes errors for optimizing the time to completion.

We note that there are still some points to improve in future work. First, we want to add to the formalization a predictive function to estimate human behavior through a realistic database that can be used in a reinforcement learning procedure. Secondly, we set in this paper the decision-making method and the strategy. We want to develop another formalization in which they will be variable and dynamically adaptable to the task.

## Data Availability

The datasets presented in this study can be found in online repositories. The names of the repository/repositories and accession number(s) can be found below: https://github.com/MelodieDANIEL/Optimizing_Human_Robot_Collaboration_Frontiers.

## Footnotes

1 Camelot Jr. is a game created by Smart Games: https://www.smartgames.eu/uk/one-player-games/camelot-jr.

2 We denote functions by lower case letters in bold, sets and subsets between braces with upper case letters in bold, indexes by lower case letters, parameters by upper case letters, and vectors (i.e., profiles) by letters in bold topped by an arrow between parenthesis.

3 The experiment protocol was approved by the ethics committee of the Clermont-Auvergne University under the number: IRB00011540-2020-48.

4 All the results are presented on https://github.com/MelodieDANIEL/Optimizing_Human_Robot_Collaboration_Frontiers.
