# Supplementary figures and images for: General Framework for the Optimization of the Human-Robot Collaboration Decision-Making Process Through the Ability to Change Performance Metrics
Source: Front Robot AI. 2021 Oct 25;8:736644. doi: 10.3389/frobt.2021.736644 (PMC8573032; doi:10.3389/frobt.2021.736644)

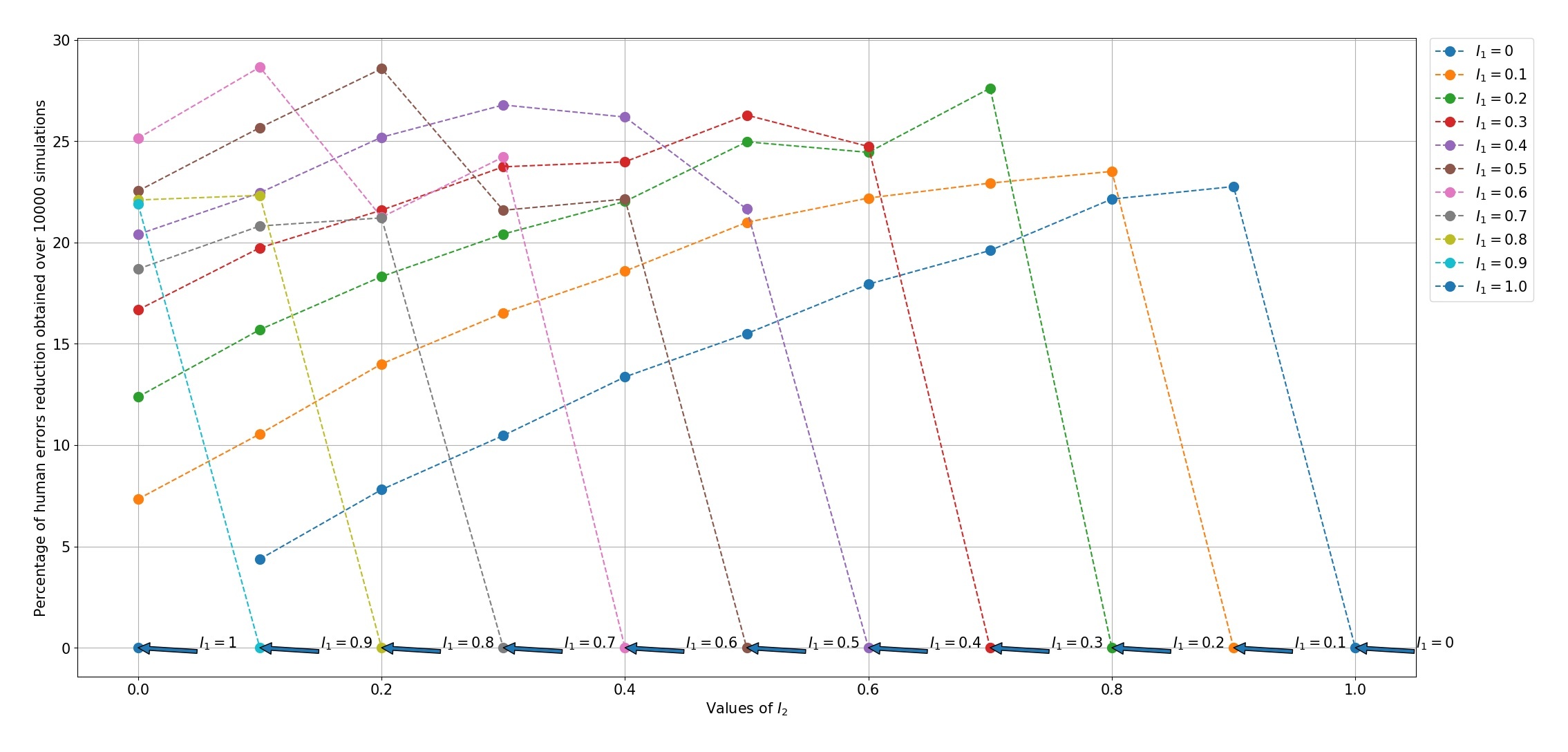

Supplement: Supplementary file 1 [file Image3.JPEG]

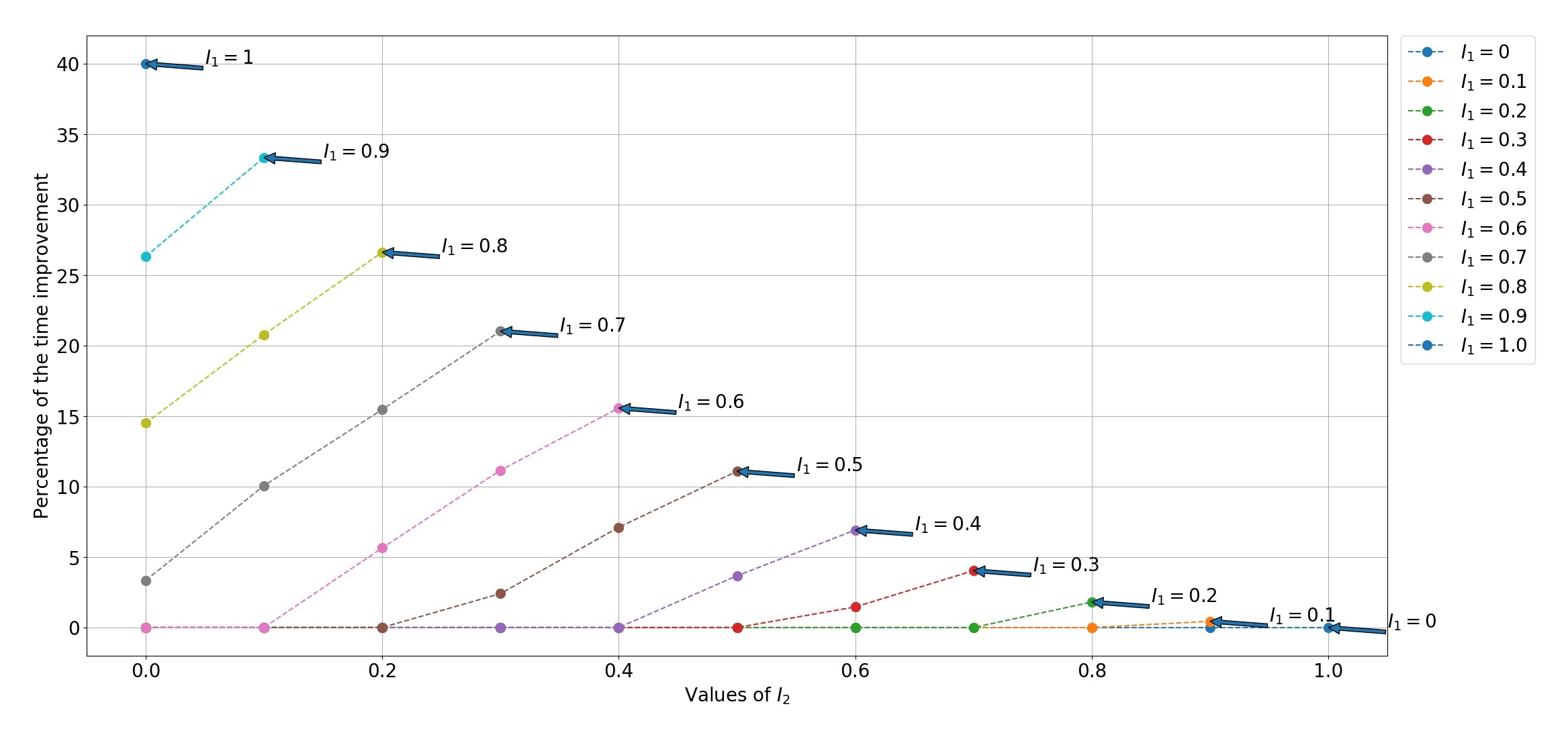

Supplement: Supplementary file 2 [file Image2.JPEG]

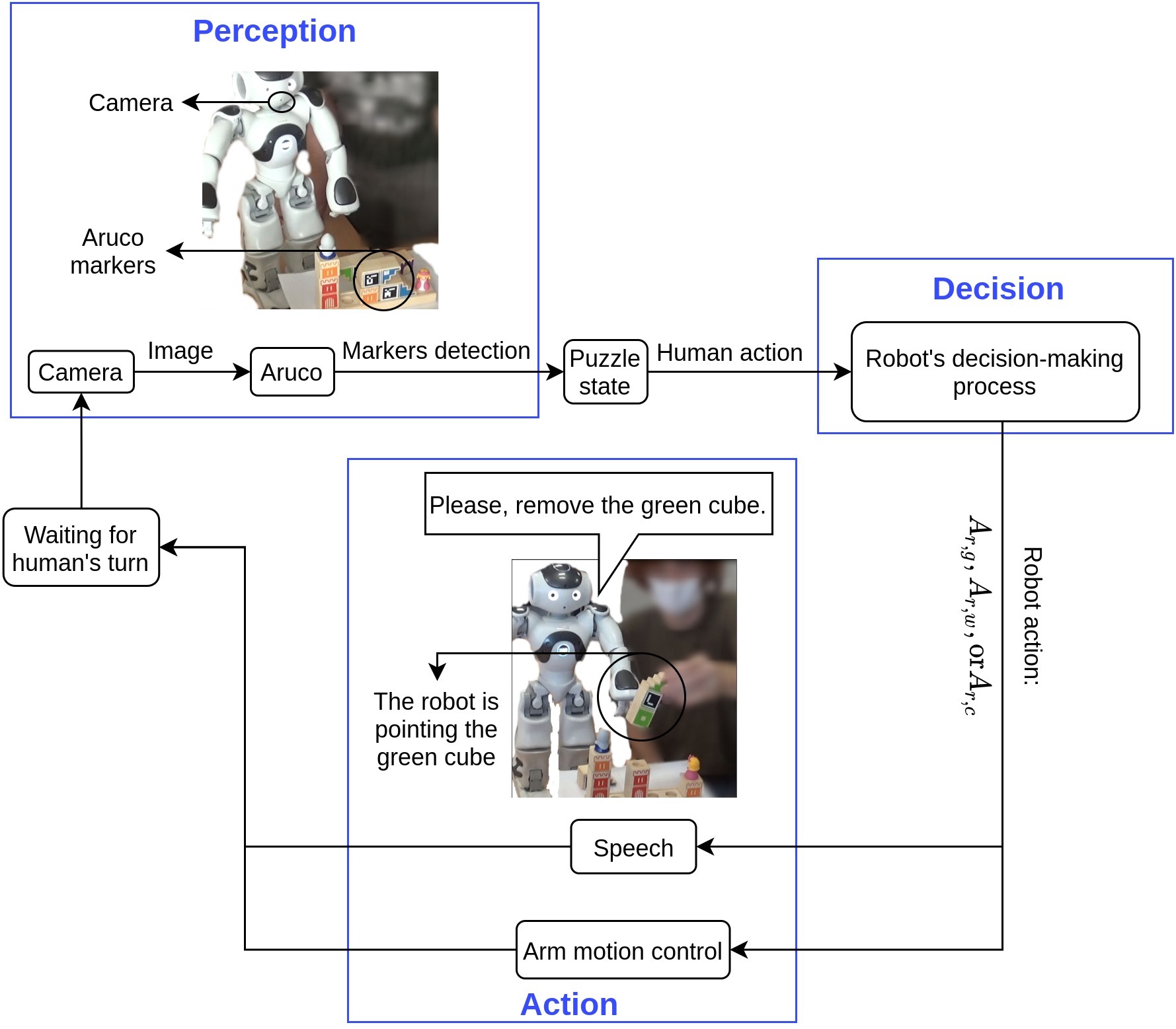

Supplement: Supplementary file 4 [file Image1.jpg]
