# Supplementary material for: General Framework for the Optimization of the Human-Robot Collaboration Decision-Making Process Through the Ability to Change Performance Metrics
Source: Front Robot AI. 2021 Oct 25;8:736644. doi: 10.3389/frobt.2021.736644 (PMC8573032; doi:10.3389/frobt.2021.736644)
Supplement: Supplementary file 3 [file DataSheet1.pdf]

Supplementary Material

1 PERFORMANCE METRICS

We present, in Table S1, the performance metrics used for the evaluation of the Human-Robot Collaboration (HRC) classified based on the task types. We define each metric according to its usage in the different task types. We also introduce some common metrics used for evaluating HRC in general rather than the performance of a specific task.

2 IMPLEMENTATION OF THE CONDUCTED EXPERIMENTS

In figure S1, we present a block diagram of our implementation of the conducted experiments. First, the participant finishes their turn. Then, it is the turn of the robot which will be mainly made up of three parts:

- Perception: Nao's camera captures the markers, then using the Aruco library (Garrido-Jurado et al., 2014), we can estimate the pose of the cubes. This allows us to compute the state of the puzzle and to identify the human action.
- Decision-making process: The robot chooses its action according to the human's one. The utility function calculates a utility for each of the robot's actions. The robot chooses, based on the Nash equilibrium, the action that has the highest utility. This is illustrated in Figure 6 of the main paper.
- Robot's action: If the robot passes its turn ($A_{r,w}$), it tells the human. In this case, the robot does not have to make any movement. On the contrary, if the robot corrects the human's action ($A_{r,c}$) or helps them by indicating where to place a cube ($A_{r,g}$), the robot will have to speak and move its arm to point out the cube to move.

3 ENTIRE EXAMPLE OF A SIMULATED TEST ON THE ASSEMBLY TASK

In the main paper, we presented the best-simulated results for illustrating the percentage of the time improvement and the percentage of the reduction of the number of human errors. In this section, we want to present both results for the same simulated test as an example. We consider a 3-cube puzzle with a ratio of the time taken by each agent (the human h and the robot r) to make an action equals to: $t_{A_h}/t_{A_r} = 1/3$. This ratio is the same as the one we had while doing the real experiment with Nao and a human participant. We define $P(A_{h,g}) = I_1$ as the probability that the human does the good action, $P(A_{h,w}) = I_2$ as the probability that the human is passing their turn and, $I_3 = 1 - (I_1 + I_2)$ as the probability that the human makes an error.

We note from Figure S2 that the percentage of the time improvement using our utility function (C_3) instead of the state-of-the-art one (C_1) for this puzzle is up to 40%. From Figure S3, the percentage of human errors reduction for the same puzzle is up to 27.9%. In both figures, each dotted line is equivalent to a specific I_1 value. Each dot corresponds to a I_2 value (read on the x-axis). For each dot knowing I_1 and I_2, we can deduce its I_3 value using $I_3 = 1 - (I_1 + I_2)$.

We have performed a lot of simulated tests, the results of which can be found on: `https://github.com/MelodieDANIEL/Optimizing_Human_Robot_Collaboration_Frontiers`.

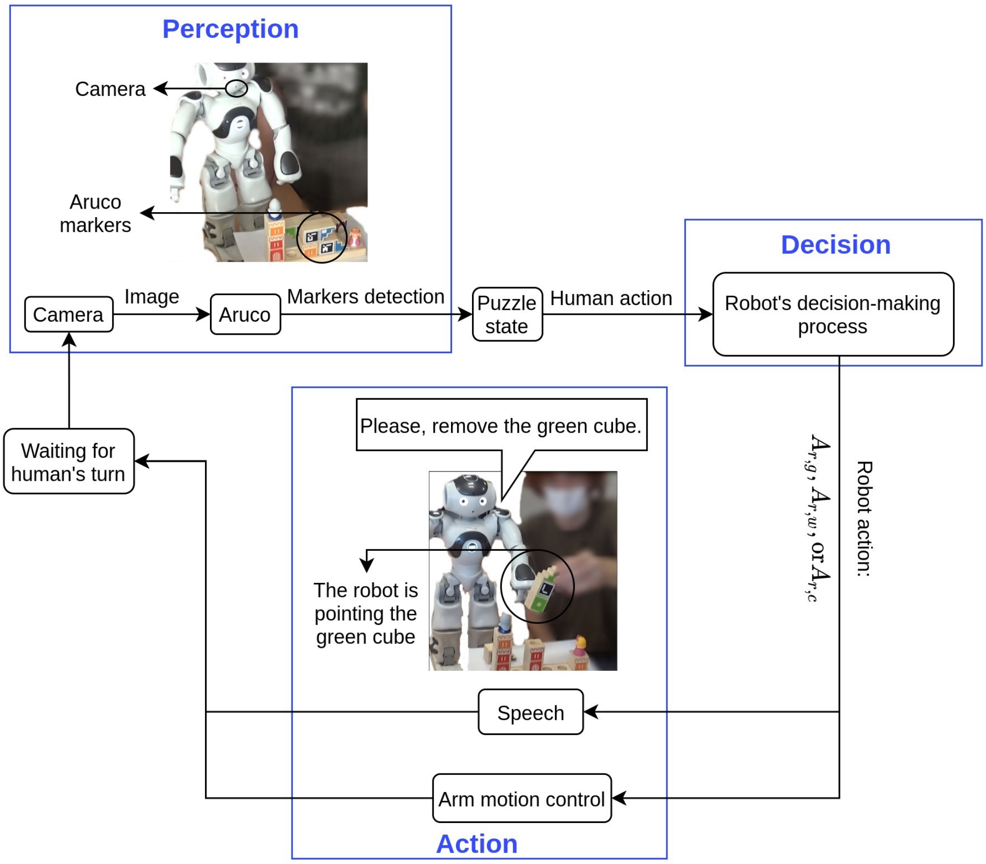

Figure S1. Implementation of the conducted experiments using ROS

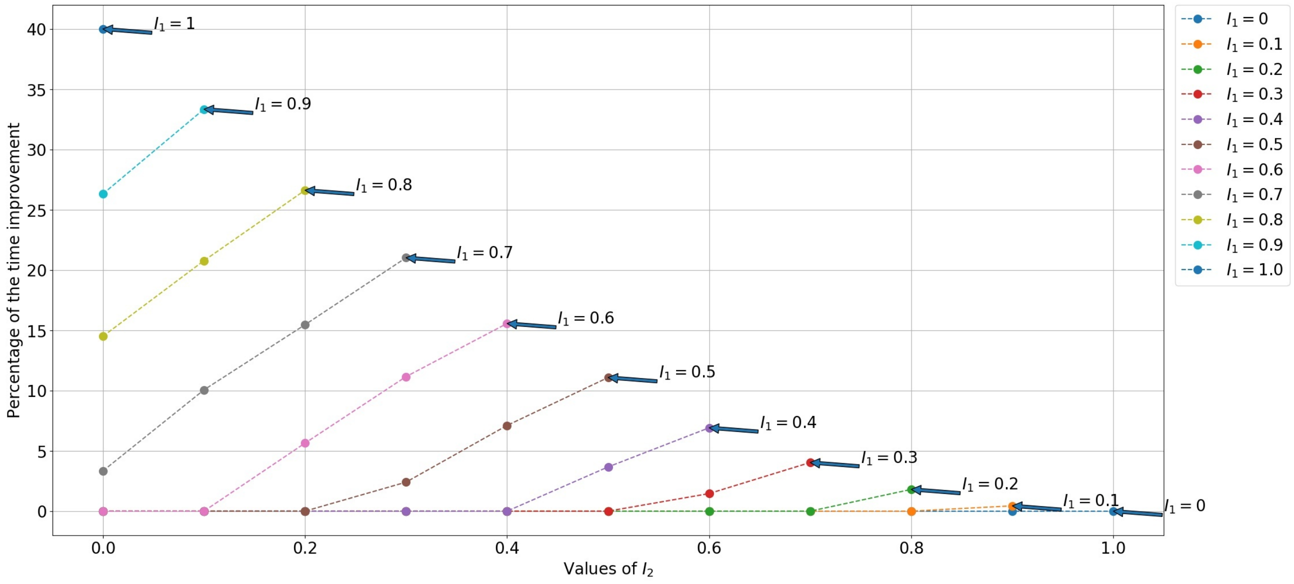

Figure S2. Percentage of time improvement between C_3 and C_1 for a 3-cube puzzle. $t_{A_h} = \{15, 0, 15\}$ and $t_{A_r} = \{45, 0, 45\}$, so the ratio $t_{A_h}/t_{A_r} = 1/3$.

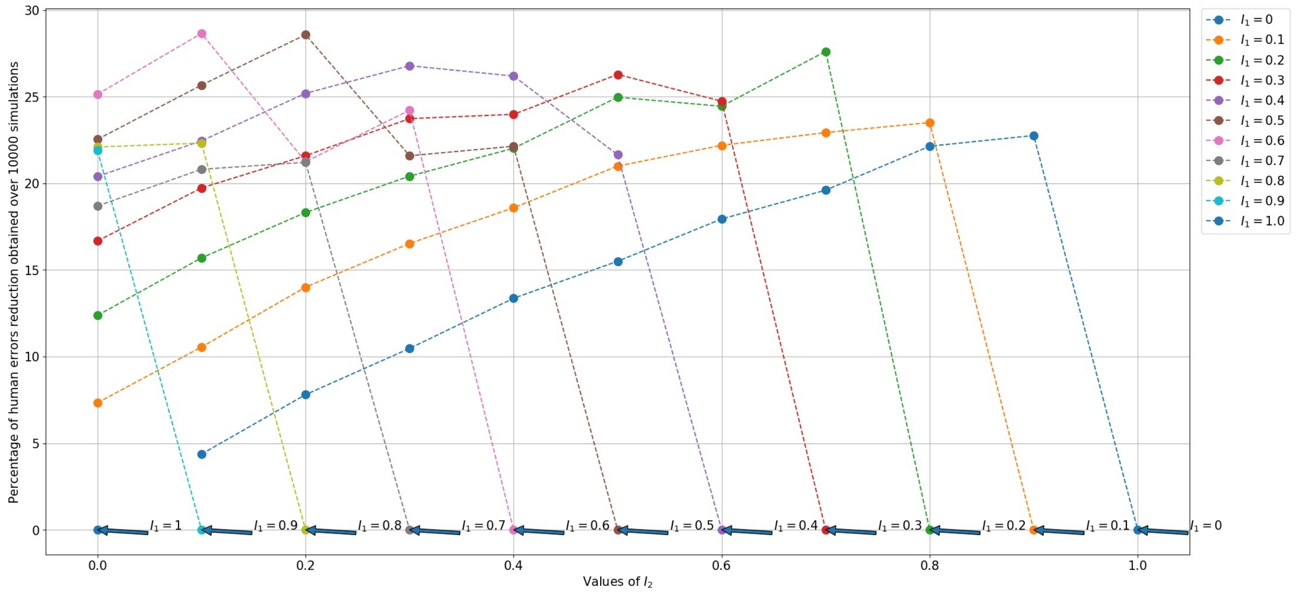

Figure S3. Percentage of human errors reduction between the predicted probability of human errors and the measured one for a 3-cube puzzle. $t_{A_h} = \{15, 0, 15\}$ and $t_{A_r} = \{45, 0, 45\}$, so the ratio $t_{A_h}/t_{A_r} = 1/3$.

4 RESULTED TABLE OF SIMULATION TESTS

In this section, we present the resulted table (Table S2) of the percentage of the time improvement and the reduction of the number of human errors for all the figures presented in the main paper and the supplementary material.

5 COMPUTATION AND EXECUTION TIME OF THE TESTS

Table S3 presents all the computation and execution times of the experiments in real and in simulation. As we can notice, the average computation time of our decision-making framework is 0.5s. This computation time is suitable for the targeted real tasks on which we want to apply this framework.

REFERENCES

Bütepage, J. and Kragic, D. (2017). Human-robot collaboration: from psychology to social robotics. *arXiv preprint arXiv:1705.10146*

Garrido-Jurado, S., Muñoz-Salinas, R., Madrid-Cuevas, F. J., and Marín-Jiménez, M. J. (2014). Automatic generation and detection of highly reliable fiducial markers under occlusion. *Pattern Recognition* 47, 2280–2292

Nelles, J., Kwee-Meier, S. T., and Mertens, A. (2018). Evaluation metrics regarding human well-being and system performance in human-robot interaction–a literature review. In *Congress of the International Ergonomics Association* (Florence, Italy, August 26-30: Springer), 124–135

Steinfeld, A., Fong, T., Kaber, D., Lewis, M., Scholtz, J., Schultz, A., et al. (2006). Common metrics for human-robot interaction. In *Proceedings of the 1st ACM SIGCHI/SIGART conference on Human-robot interaction* (Salt Lake City, Utah, USA, March 2-3), 33–40

Task	Performance metrics	Definition or usability
Navigation	Failure rate	Percentage of navigation tasks completion failure
	Accuracy	The accuracy of the navigation
	Ergonomy or posture	Human ergonomy or posture
	Time to completion	The time needed to complete the task
	Rapidity	The time needed by the robot to adapt itself to the human or vice-versa
Perception	Velocity	The speed of the perception of the robot
	Accuracy	The accuracy of the navigation
	Time to completion	The time needed to complete the task
	Fluency	The fluency of the perception
	Effectiveness	Percentage of the success of the robot's perception
	Number of errors	The number of failures in the robot's perception
Management	Time delivery	The time needed to deliver the request from the robot to the human
	Time request	The time needed by the human (operator) to notice the request
	Number of human errors	The number of times the human cannot identify the situation with awareness
	Number of robot errors	The number of times the robot is misinterpreting human desires
	Trust	Trust of the human in the robot
	Number of actions	The number of actions needed to accomplish the task from the human and the robot
	Cognitive load	The workload required for the human to adapt to the robot
Manipulation	Positional accuracy	The accuracy of the position reached by the robot
	Positional repeatability	The repeatability of the robot to reach the same position
	Velocity	The speed of the robot to do the manipulation
	Time to completion	The time needed to complete the task
	Rapidity	The time needed by the robot to adapt itself to the human or vice-versa
	Cognitive load	The workload required for the human to adapt to the robot
	Ergonomy or posture	Human ergonomy or posture
	Dexterity	The robot's dexterity in doing the manipulation
	Effort or force	The physical effort (or force) that the human must provide to perform the manipulation
	Number of human errors	The number of times the human cannot identify the situation with awareness
	Number of robot errors	The number of times the robot is misinterpreting human desires
	Number of actions	The number of actions needed to accomplish the task from the human and the robot
Social	Persuasiveness	The ability of the robot to persuade the human about something
	Trust	Trust of the human in the robot
	Engagement in social characteristics	Engagement in social characteristics such as emotion, dialogue, personality. The engagement can be measured through the robot's acquisition time for capturing human attention and the duration of holding human interest
	Compliance	The compliance of the robot in appearance, adherence to norms, etc.
Common metrics	Effectiveness	The percentage of the mission that was accomplished with the designed autonomy
	Time to completion	The time needed to complete the task
	Number of human errors	The number of times the human cannot identify the situation with awareness
	Number of robot errors	The number of times the robot is misinterpreting human desires
	Number of actions	The number of actions needed to accomplish the task from the human and the robot
	Cognitive load	The workload required for the human to adapt to the robot
	Self-awareness	The robot knows its accuracy
	Autonomy	The robot autonomy

Table S1. Some metrics considered for the evaluation of HRC classified based on the task types (Steinfeld et al., 2006; Bütepage and Kragic, 2017; Nelles et al., 2018)

	Percentage of time improvement		Percentage of human errors reduction	
I_1, I_2, and I_3 values	Figure 6 (main paper)	Figure S2	Figure 7 (main paper)	Figure S3
$I_1 = 0$, $I_2 = 0.1$, and $I_3 = 0.9$	0.0	0.0	7.09412331431	4.37067607062
$I_1 = 0$, $I_2 = 0.2$, and $I_3 = 0.8$	0.0	0.0	12.6336858252	7.8020796966
$I_1 = 0$, $I_2 = 0.3$, and $I_3 = 0.7$	0.0	0.0	16.556882222	10.4704561576
$I_1 = 0$, $I_2 = 0.4$, and $I_3 = 0.6$	0.0	0.0	19.2303631915	13.3638679771
$I_1 = 0$, $I_2 = 0.5$, and $I_3 = 0.5$	0.0	0.0	22.3643444888	15.4993421616
$I_1 = 0$, $I_2 = 0.6$, and $I_3 = 0.4$	0.0	0.0	24.8542177267	17.9408050283
$I_1 = 0$, $I_2 = 0.7$, and $I_3 = 0.3$	0.0	0.0	28.4374851075	19.6044166944
$I_1 = 0$, $I_2 = 0.8$, and $I_3 = 0.2$	0.0	0.0	31.2453571429	22.1412554113
$I_1 = 0$, $I_2 = 0.9$, and $I_3 = 0.1$	0.0	0.0	29.2866666667	22.7585714286
$I_1 = 0$, $I_2 = 1$, and $I_3 = 0$	0.0	0.0	0.0	0.0
$I_1 = 0.1$, $I_2 = 0$, and $I_3 = 0.9$	0.0	0.0	17.5302972429	7.33482163197
$I_1 = 0.1$, $I_2 = 0.1$, and $I_3 = 0.8$	0.0	0.0	19.2143318311	10.5479301203
$I_1 = 0.1$, $I_2 = 0.2$, and $I_3 = 0.7$	0.0	0.0	21.3374721068	14.0052043345
$I_1 = 0.1$, $I_2 = 0.3$, and $I_3 = 0.6$	0.0	0.0	23.5786203979	16.5280674286
$I_1 = 0.1$, $I_2 = 0.4$, and $I_3 = 0.5$	0.0	0.0	26.7249188197	18.5858323843
$I_1 = 0.1$, $I_2 = 0.5$, and $I_3 = 0.4$	0.0	0.0	27.5937682456	20.9918095099
$I_1 = 0.1$, $I_2 = 0.6$, and $I_3 = 0.3$	0.0	0.0	28.9937657713	22.1913226588
$I_1 = 0.1$, $I_2 = 0.7$, and $I_3 = 0.2$	0.0	0.0	32.0019444444	22.9278030303
$I_1 = 0.1$, $I_2 = 0.8$, and $I_3 = 0.1$	0.0	0.0	34.205	23.5061904762
$I_1 = 0.1$, $I_2 = 0.9$, and $I_3 = 0$	0.646808142428	0.441773745339	0.0	0.0
$I_1 = 0.2$, $I_2 = 0$, and $I_3 = 0.8$	0.0	0.0	25.4460206798	12.376411145
$I_1 = 0.2$, $I_2 = 0.1$, and $I_3 = 0.7$	0.0	0.0	25.6904326925	15.6962303954
$I_1 = 0.2$, $I_2 = 0.2$, and $I_3 = 0.6$	0.0	0.0	27.5106709889	18.315465336
$I_1 = 0.2$, $I_2 = 0.3$, and $I_3 = 0.5$	0.0	0.0	30.0794412809	20.4125719276
$I_1 = 0.2$, $I_2 = 0.4$, and $I_3 = 0.4$	0.0	0.0	30.8272285354	22.0221180209
$I_1 = 0.2$, $I_2 = 0.5$, and $I_3 = 0.3$	0.0	0.0	31.9211207311	24.9664681615
$I_1 = 0.2$, $I_2 = 0.6$, and $I_3 = 0.2$	0.0	0.0	34.119047619	24.4444642857
$I_1 = 0.2$, $I_2 = 0.7$, and $I_3 = 0.1$	0.765528401311	0.0	0.0	27.6111904762
$I_1 = 0.2$, $I_2 = 0.8$, and $I_3 = 0$	2.4788012545	1.80659141302		0.0
$I_1 = 0.3$, $I_2 = 0$, and $I_3 = 0.7$	0.0	1.91789786313	30.2540826341	16.6733556418
$I_1 = 0.3$, $I_2 = 0.1$, and $I_3 = 0.6$	0.0	0.0	30.7411696561	19.7313028361
$I_1 = 0.3$, $I_2 = 0.2$, and $I_3 = 0.5$	0.0	0.0	31.3673661689	21.5925174216
$I_1 = 0.3$, $I_2 = 0.3$, and $I_3 = 0.4$	0.0	0.0	33.7247655123	23.7318043068
$I_1 = 0.3$, $I_2 = 0.4$, and $I_3 = 0.3$	0.0	0.0	34.3449001924	23.9794936545
$I_1 = 0.3$, $I_2 = 0.5$, and $I_3 = 0.2$	0.0	0.0	36.4016269841	26.2835119048
$I_1 = 0.3$, $I_2 = 0.6$, and $I_3 = 0.1$	2.77628815301	1.46088929863	36.7869047619	24.7346428571
$I_1 = 0.3$, $I_2 = 0.7$, and $I_3 = 0$	5.6292792232	4.03759880367	0.0	0.0
$I_1 = 0.4$, $I_2 = 0$, and $I_3 = 0.6$	0.0	0.0	36.3891268668	20.3970746112
$I_1 = 0.4$, $I_2 = 0.1$, and $I_3 = 0.5$	0.0	0.0	35.2710778111	22.4513601676
$I_1 = 0.4$, $I_2 = 0.2$, and $I_3 = 0.4$	0.0	0.0	35.8305131674	25.1917275086
$I_1 = 0.4$, $I_2 = 0.3$, and $I_3 = 0.3$	0.0	0.0	36.4988095238	26.7846236171
$I_1 = 0.4$, $I_2 = 0.4$, and $I_3 = 0.2$	2.97937356761	0.0	37.8678571429	26.1883928571
$I_1 = 0.4$, $I_2 = 0.5$, and $I_3 = 0.1$	6.8298290148	3.67128494973	35.0916666667	21.646547619
$I_1 = 0.4$, $I_2 = 0.6$, and $I_3 = 0$	10.0581040567	6.91767350379	0.0	0.0
$I_1 = 0.5$, $I_2 = 0$, and $I_3 = 0.5$	0.0	0.0	38.5427888223	22.5377313961
$I_1 = 0.5$, $I_2 = 0.1$, and $I_3 = 0.4$	0.0	0.0	39.8677200577	25.6673357198
$I_1 = 0.5$, $I_2 = 0.2$, and $I_3 = 0.3$	2.5889362939	0.0	40.2805687831	28.5753607504
$I_1 = 0.5$, $I_2 = 0.3$, and $I_3 = 0.2$	7.56507185318	2.4071413430	38.0336309524	21.5879166667
$I_1 = 0.5$, $I_2 = 0.4$, and $I_3 = 0.1$	12.187798206	7.11099379702	41.565	22.1403571429
$I_1 = 0.5$, $I_2 = 0.5$, and $I_3 = 0$	15.7060720797	11.0874137267	0.0	0.0
$I_1 = 0.6$, $I_2 = 0$, and $I_3 = 0.4$	0.0	0.0	41.0730555556	25.1399181374
$I_1 = 0.6$, $I_2 = 0.1$, and $I_3 = 0.3$	8.17205250781	0.0	42.012965368	28.6511640212
$I_1 = 0.6$, $I_2 = 0.2$, and $I_3 = 0.2$	14.1780122742	5.64235894103	43.109047619	21.2425054113
$I_1 = 0.6$, $I_2 = 0.3$, and $I_3 = 0.1$	18.8995795602	11.1593671564	45.3266666667	24.2171428571
$I_1 = 0.6$, $I_2 = 0.4$, and $I_3 = 0$	23.2260934025	15.568227852	0.0	0.0
$I_1 = 0.7$, $I_2 = 0$, and $I_3 = 0.3$	17.2439908187	3.33567251462	44.296547619	18.6905624931
$I_1 = 0.7$, $I_2 = 0.1$, and $I_3 = 0.2$	22.4988118634	10.0605063426	45.0121428571	20.8104816017
$I_1 = 0.7$, $I_2 = 0.2$, and $I_3 = 0.1$	27.4951742932	15.4641925539	43.6866666667	21.2117857143
$I_1 = 0.7$, $I_2 = 0.3$, and $I_3 = 0$	31.8854548846	21.023549533	0.0	0.0
$I_1 = 0.8$, $I_2 = 0$, and $I_3 = 0.2$	33.0590564877	14.5036859249	44.6876190476	22.0960714286
$I_1 = 0.8$, $I_2 = 0.1$, and $I_3 = 0.1$	37.9880475163	20.7778656126	45.2483333333	22.3210714286
$I_1 = 0.8$, $I_2 = 0.2$, and $I_3 = 0$	41.9390153589	26.6215414675	0.0	0.0
$I_1 = 0.9$, $I_2 = 0$, and $I_3 = 0.1$	49.8353835563	26.317921026	50.5583333333	21.8939285714
$I_1 = 0.9$, $I_2 = 0.1$, and $I_3 = 0$	53.3069306931	33.3469782673	0.0	0.0
$I_1 = 1$, $I_2 = 0$, and $I_3 = 0$	66.6666666667	40.0	0.0	0.0

Table S2. Time improvement percentage and human errors reduction percentage obtained for all the figures of the main paper and the supplementary material.

	Step	Time in seconds
Real tests	Computation time of the decision-making (applying our formalization)	The robot takes an average of 0.5s to choose the action to perform after knowing the state of the puzzle through the perception part.
	Time taken by the robot for the perception of the puzzle state	The robot takes between 20s and 30s depending on how well the cubes are placed and how many cubes are left to assemble.
	Time taken by the robot for doing a physical movement	The robot takes on average 15s for doing a physical movement.
	Waiting time for the robot when it gives an indication for the human	The robot waits between 5s and 15s each time it gives an indication to the human, depending on its complexity (for example, to ask the human to remove a cube, the robot waits 5s, and to ask the human to take a certain cube and place it in a certain position, the robot waits 15s).
	Global time taken by the robot to perform an action	It is between 20s and 60s, depending on the complexity of the movement (the number of cubes left to assemble at this iteration) and if the robot gives indications to the human. We considered that it was 60s.
	Global time taken by the human to perform an action	The human takes between 1s and 30s, depending on the complexity of the movement (if they know what to do or not). We considered that it was 20s.
Tests in simulation	Time required for all probability distributions of possible human actions without printing the figures (such as Figures S2 and S3)	The Python code takes between 80s and 100s on a Dell laptop with an Intel Core i7 CPU and 32GB RAM.

Table S3. Computation and execution times of the experiments in real and in simulation
